# Enrichment of horizontally transferred gene clusters in bacterial extracellular vesicles via non lytic mechanisms

**DOI:** 10.1093/ismejo/wraf193

**Published:** 2025-08-29

**Authors:** Sotaro Takano, Satoshi Takenawa, Divya Naradasu, Kangmin Yan, Xinxin Wen, Tomoko Maehara, Nobuhiko Nomura, Nozomu Obana, Masanori Toyofuku, Michihiko Usui, Wataru Ariyoshi, Akihiro Okamoto

**Affiliations:** Research Center for Macromolecules and Biomaterials, National Institute for Materials Science, 1-1 Namiki, Tsukuba, Ibaraki 305-0044, Japan; Integrated Bioresource Information Division, BioResource Research Center, RIKEN, 3-1-1 Koyadai, Tsukuba, Ibaraki 305-0074, Japan; Research Center for Macromolecules and Biomaterials, National Institute for Materials Science, 1-1 Namiki, Tsukuba, Ibaraki 305-0044, Japan; Research Center for Macromolecules and Biomaterials, National Institute for Materials Science, 1-1 Namiki, Tsukuba, Ibaraki 305-0044, Japan; Research Center for Macromolecules and Biomaterials, National Institute for Materials Science, 1-1 Namiki, Tsukuba, Ibaraki 305-0044, Japan; Research Center for Macromolecules and Biomaterials, National Institute for Materials Science, 1-1 Namiki, Tsukuba, Ibaraki 305-0044, Japan; Research Center for Macromolecules and Biomaterials, National Institute for Materials Science, 1-1 Namiki, Tsukuba, Ibaraki 305-0044, Japan; Graduate School of Life and Environmental Sciences, University of Tsukuba, 1-1-1 Tennodai, Tsukuba, Ibaraki 305-8577, Japan; Microbiology Research Center for Sustainability, University of Tsukuba, 1-1-1 Tennodai, Tsukuba, Ibaraki 305-8577, Japan; Microbiology Research Center for Sustainability, University of Tsukuba, 1-1-1 Tennodai, Tsukuba, Ibaraki 305-8577, Japan; Transborder Medical Research Center, Faculty of Medicine, University of Tsukuba, University of Tsukuba, 1-1-1 Tennodai, Tsukuba, Ibaraki 305-8577, Japan; Graduate School of Life and Environmental Sciences, University of Tsukuba, 1-1-1 Tennodai, Tsukuba, Ibaraki 305-8577, Japan; Microbiology Research Center for Sustainability, University of Tsukuba, 1-1-1 Tennodai, Tsukuba, Ibaraki 305-8577, Japan; Division of Periodontology, Department of Oral Function, Kyushu Dental University, 2 Chome-6-1 Manazuru, Kokurakita Ward, Kitakyushu, Fukuoka 803-8580, Japan; Division of Infection and Molecular Biology, Department of Health Promotion, Kyushu Dental University, 2 Chome-6-1 Manazuru, Kokurakita Ward, Kitakyushu, Fukuoka 803-8580, Japan; Research Center for Macromolecules and Biomaterials, National Institute for Materials Science, 1-1 Namiki, Tsukuba, Ibaraki 305-0044, Japan; Graduate School of Life and Environmental Sciences, University of Tsukuba, 1-1-1 Tennodai, Tsukuba, Ibaraki 305-8577, Japan; Graduate School of Chemical Sciences and Engineering, Hokkaido University, North 13 West 8, Kita-ku, Sapporo, Hokkaido 060-8628, Japan; Research Center for Autonomous Systems Materialogy, Institute of Innovative Research, Tokyo Institute of Technology, 4259 Nagatsuta-cho, Midori-ku, Yokohama, Kanagawa 226-8501, Japan

**Keywords:** membrane vesicles, horizontal gene transfer, oral microbiota, CRISPR-Cas, O-antigen gene cluster, Porphyromonas gingivalis

## Abstract

Bacterial extracellular vesicles are emerging as key mediators of horizontal gene transfer, enhancing microbial adaptability. A critical factor determining the effectiveness of horizontal gene transfer is the fraction of vesicles containing specific functional genes. However, the proportion of containing specific DNA fragments has not been adequately determined, which hinders the understanding of the conditions and mechanisms that facilitate the incorporation of specific genes into the vesicles and possible evolutionary roles of vesicle-derived DNA. Here, we demonstrate that enrichment of horizontally transferred genes into bacterial extracellular vesicles is driven by cellular processes by profiling the DNA content of hundreds of individual vesicles using a microdroplet-based sequencing technique. This approach revealed unique DNA profiles in vesicles from the oral pathogen *Porphyromonas gingivalis*, pinpointing genomic regions related to DNA reorganization such as CRISPR-Cas clusters. Comparative genomic and phylogenetic analyses of *Porphyromonas* genomes revealed traces of horizontal gene transfer in vesicle-enriched genes. Modulating vesicle-biogenesis routes, quantitative real-time PCR revealed that this selective enrichment was driven by blebbing-driven DNA packaging mechanisms rather than stress-induced lysis. Applied to dental plaque-derived bacterial extracellular vesicles, the droplet-based approach reveled O-antigen biosynthetic genes, key for host–bacterial interactions, were prevalent in the vesicles from *Alcaligenes faecalis*, suggesting the vesicles from this bacterium can modulate pathogenicity in oral biofilms through targeted DNA packaging. These findings suggest the prevalence of functionally relevant gene clusters in bacterial extracellular vesicles in oral microbiota and their evolutionary roles as DNA cargoes for modulating phage–bacterial and host–bacterial interactions via horizontal gene transfer.

## Introduction

Horizontal gene transfer (HGT), a fundamental mechanism of microbial evolution [1], enables bacterial cells to acquire novel phenotypic traits (e.g. antibiotic resistance), fostering rapid adaptations to environmental challenges [2–4]. Besides ensuring individual survival, HGT can also contribute to genetic diversification within microbial populations [5]. This allows the sharing and integration of beneficial genes across species barriers [6, 7], catalyzing the emergence of novel capabilities within bacterial communities [2, 8].

Bacterial extracellular vesicles (BEVs), nano-vehicles of diverse biomolecules produced by both Gram-positive and Gram-negative bacteria in various habitats [9, 10], have emerged as critical facilitators of HGT [11, 12]. A recent study focusing on BEVs in marine environment revealed the prevalence of mobile genetic elements in BEV-incorporated DNA, supporting its significance as DNA cargo [13]. The molecules inside BEVs (e.g. DNA) are generally condensed, secreted, and protected from the risks of degradation [14, 15], increasing the success of gene transfer compared with uptake of extracellular DNA (e.g. natural transformation) [16]. BEVs have the potential to transfer genetic material not only within a species but also across phylogenetically distant species [16–18]. These characteristics are distinct from other molecular mechanisms (e.g. conjugation and natural transformation), suggesting the unique role of BEVs for expanding the genetic repertoire and adaptability of microbial communities.

Despite their potential evolutionary significance via HGT, the origin, and nature of the genetic material within BEVs remains largely underexplored. For instance, although certain genes appear to be selectively enriched in BEVs and amount or regions of packaged DNA vary depending on bacterial conditions or BEV types [19–24], the mechanisms behind the selective incorporation of specific DNA remain unclear. In contrast, existing models suggest that DNA incorporation into BEVs occurs randomly during bacterial cell death [14], abolishing the idea of selective DNA packaging. Thus, whether BEV-mediated HGT plays a role in bacterial evolution through specific DNA packaging mechanisms, rather than being purely stochastic, remains a subject of debate.

A critical factor determining the effectiveness of HGT is the quantity of BEVs containing specific genes [17], underscoring the need to understand the genes present and the diversity of the DNA encapsulated in individual BEVs. However, identifying and characterizing DNA within BEVs pose significant challenges, as conventional metagenomic methods cannot adequately differentiate DNA variations among individual vesicles. To address these limitations, we applied a single-nanoparticle droplet DNA sequencing (NP-DS) technique, adapted from single-cell genomic methodologies [25–27]. The genetic content of individual vesicles was profiled by applying *P. gingivalis*—and human dental plaque-derived BEVs. Given that this organism and its BEVs are implicated in periodontitis and systemic diseases [28, 29], our study highlights the specific features of BEV-derived DNA that may play important roles in microbial interactions, particularly within oral biofilms and infections.

## Materials and methods

### Isolation and purification of bacterial extracellular vesicles from *P. gingivalis*

*P. gingivalis* W83 was grown in Gifu Anaerobic Medium at 37°C. To maintain anaerobic conditions, 20 min of N_2_/CO_2_ (80,20 v/v) gas sparging was performed prior to culture inoculation. The culture was grown at 37°C until the OD_600_ reached over ≈0.6, followed by centrifugation for 10 min at 7800 rpm and 4°C. The supernatant was passed through 0.22 μm filters to remove any cell debris and then ultracentrifuged for 2 h at 200,000 × *g* at 4°C, and the resulting pellets were resuspended in phosphate-buffered saline (PBS).

### DNase I treatment

DNase I (13 units (U) / μl) (NIPPON GENE) was added to a final concentration of 2 U to treat the external DNA of nanoparticles. The sample was stored at 37°C for 30 min, followed by DNase I deactivation by heating the sample at 80°C for 10 min. We confirmed that same amount of DNase I treatment can degrade the control DNA samples (200 ng of pUC19 plasmid) using the same procedure.

### DNA isolation and sequencing of bulk bacterial extracellular vesicle samples

DNA from DNase I-treated BEV samples was extracted and purified using an Isoplant-II DNA extraction kit (NIPPON GENE) according to the manufacturer’s instructions. The total amount of extracted DNA was quantified using a Qubit 1X dsDNA High Sensitivity Assay Kit (Thermo Fisher). Sequencing libraries were prepared using the QIAseq FX DNA Library Kit (QIAGEN) according to the manufacturer’s protocols and sequenced using the NextSeq 2000 System (Illumina) with a 2 × 150 bp configuration. The obtained fastq files were processed using fastp 0.19.5 [30], and the sequence reads were aligned to the assembly genomes of the host bacterium (*P. gingivalis*: GCF_000007585.1) using bowtie2 [31] with option (−sensitive-local).

### Nanoparticle tracking analysis

NTA was performed using a ZetaView (Particle Metrix) to quantify the nanoparticles. The samples were measured by scanning 11 cell positions and capturing images at 30 fps. For measurements using fluorescent signals, 488/500 nm and 660/680 nm laser-filter units were used with the following camera settings: sensitivity: 70–80; shutter: 200; minimum trace length: 15. For measurements in scatter mode, we used the following settings: sensitivity: 65; shutter: 200; minimum trace length: 15. The captured video images were further analyzed using the ZetaView Software. We confirmed that measuring PBS only with lipid-dye did not harbor any detectable signal and the detected particles were stained nanoparticles and not dye-aggregates.

### Droplet genome sequencing

Droplet genome sequencing was performed by bitBiome (Japan), as previously described [32]; the details are described in the Supplemental Methods. Briefly, the cell or nanoparticle suspensions were mixed with 1.5% agarose solutions to yield an expected percentage of positive droplets of 40% [25]. For the cell-DS, the encapsulated cells were lysed using lysis solutions in gel beads. The droplets in the cell-DS and NP-DS were processed for multiple displacement analysis using the REPLI-g Single Cell Kit (QIAGEN) and the beads were stained with 1× SYBR Green (Thermo Fisher Scientific). Green fluorescence-positive beads were sorted using a FACSMelody cell sorter (BD Biosciences). The collected positive droplets proceeded to the second round of whole-genome amplification using the REPLI-g Single Cell Kit. Whole-genome amplified samples with sufficient DNA were further subjected to whole-genome sequencing analysis using the Nextera XT DNA Library Prep Kit (Illumina) according to the manufacturer’s protocols and sequenced using HiSeq System (Illumina).

### Subjects and dental plaque sample collection for droplet sequencing

Patients were recruited with periodontitis, classified as having generalized stage III-grade C [33]. These patients had not taken antimicrobials within the previous 3 months, were non-smokers, or had diabetes or any other serious systemic disease. The present study was approved by the ethics committee of Kyushu Dental University (ethical approval number: 26–28) and conducted in accordance with the approved guidelines. All the participants provided written informed consent.

Dental plaque biofilm samples (*n* = 3) were collected as follows. Rolled cotton was placed next to the teeth to prevent saliva contamination. Supra- and subgingival plaques were collected without blebbing by a periodontist using a curette. The collected dental plaque biofilm samples were immediately resuspended in 1 ml of PBS. Thereafter, the biofilm was treated with 100 μg/ml Proteinase K (QIAGEN) for 1 h at 37°C with vortexing at 20-min intervals to degrade extracellular matrices, and the treated sample was centrifuged for 10-min at 6000 × *g* at room temperature. The resulting pellet was resuspended into OMNIgene-ORAL OM-501 (DNA Genotek) and used for cell-DS. The supernatant was passed through 0.22 μm filters to remove any extracellular substrates. The filtered supernatant was ultracentrifuged for 2 h at 200,000 × *g* at 4°C, and the resulting pellets were re-suspended in 10 mM HEPES with 0.85% NaCl for NP-DS.

### Data analysis in NP-DS and cell-DS

The paired-end read sequences from each droplet were first processed using fastp 0.19.5 and then assembled using SPAdes 3.12.0. Coding sequences (CDSs), rRNAs, and tRNAs were extracted from the contigs using Prokka 1.14.6 [34]. Extracted CDSs were grouped and clustered using CD-HIT. Clustered CDSs were first annotated by a homology search against the National Center for Biotechnology Information (NCBI) nonredundant (nr) database (downloaded on July 1, 2021) using DIAMOND 2.0.8 [35]. For each CDS, the GenBank accession number of the best-hit protein sequence was used for taxonomic annotation with BASTA [36]. The CDSs classified as Bacterial at the kingdom-level were further annotated by a homology search against protein sequences in the genome taxonomy database [37] (version r202) by DIAMOND stricter taxonomic annotation, with a threshold in a percentage identity ≥80%.

The bacterial taxa classified as potential contaminants in low-biomass human samples in previous studies were eliminated [38, 39] (see Supplementary Data 4). For droplets determined to be BEV-containing, the CDSs were first grouped by GTDB accession number and the total CDS length assigned to each GTDB taxonomy was calculated. Thereafter, the most abundantly detected GTDB taxonomy was designated to the MFT (the most frequently detected taxon) for each droplet. For details of the computational analysis, see Supplementary Methods.

## Results

### Enrichment of specific genomic regions in *P. gingivalis*-derived BEVs

**Figure 1:**
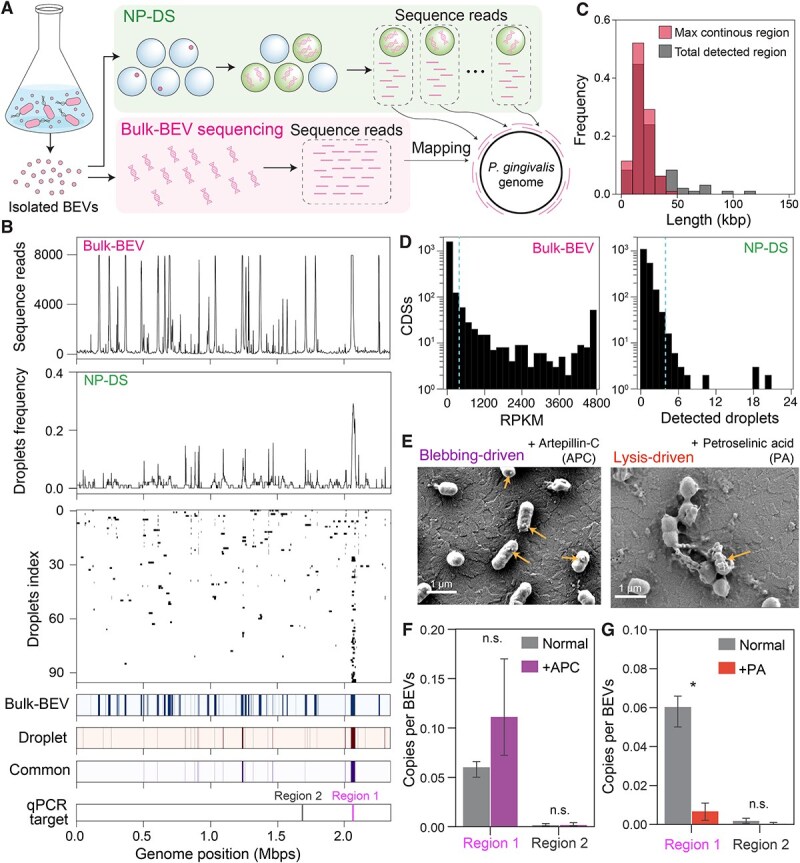
Enrichment of DNA at specific loci of the host genome in *P. gingivalis* BEVs. (A) A schematic of the analysis. Using the isolated bacterial extracellular vesicles (BEVs) from the bacterial culture, we performed nanoparticle droplet sequencing (NP-DS) by DNA amplification of encapsulated droplets or bulk-BEV sequencing by directly extracting DNA from the whole BEV particles for shotgun sequencing. In NP-DS, the collected sequence reads in each droplet were mapped to the original bacterial genome to obtain the mapping profiles of individual droplets. In bulk-BEV sequencing, all sequence reads were collectively mapped to the genome. In both cases, we analyzed BEV samples derived from a single culture medium. (B) Loci of mapped regions of sequence reads in BEVs on the original bacterial chromosomes. The number of mapped reads in the next-generation sequencing of the bulk-BEV sample is displayed in the top panel. In the second top panel, the frequency of positive droplets among 96 droplets across each bacterial genome is shown. In the third top panel, the detected genomic regions in all 96 droplets are displayed vertically. In each row, the positions on the genome where the sequence reads were mapped in each droplet are indicated with filled areas. The genomic regions determined as significantly enriched in bulk-BEVs (more than 75th percentile +2*IQR (interquartile range)) or droplet sequencing (binomial test, *P* < 0.01) are indicated with filled areas in the bottom panels. The enriched chromosomal regions in both the droplet and bulk-BEVs sequence analysis are indicated with filled areas (in the bottom-most panel). (C) The distributions of the total lengths (enclosed by solid lines) or the longest contiguous genomic region (enclosed by dashed lines) mapped by sequence reads in each droplet (*n* = 96). (D) Abundance distribution of 1873 CDSs in BEVs. In the bulk-BEV sequencing, the abundance was estimated according to the frequency of mapped reads (RPKM). In the case of the NP-DS, the number of droplets detected was used as a metric of the abundance of each CDS. Dashed lines indicate the threshold for enriched CDSs (i.e. outliers, 75 percentile +2*IQR (interquartile range)). (E) SEM images of *P. gingivalis* cells treated with artepillin-C or petroselinic acid. BEV productions are indicated by arrows. (F), (G) DNA copy numbers of the target genomic regions in the BEV samples quantified using qPCR when the cells were treated with artepillin-C (APC, panel F) or petroselinic acid (PA, panel G). We targeted two representative regions: region 1 was significantly enriched in the NP-DS and region 2 was not detected in any of the 96 droplets (those target sites were labelled as bars in Fig. 1B, respectively). Here, the copy numbers per particle of each genomic region under two different conditions are displayed. Error bars indicate the standard deviation in triplicate experiments. Asterisks show statistical significance levels according to the Student’s *t-*test (*: *P* < 0.05, n.s.: not significant).

We explored the characteristics of DNA sequences in BEVs produced by *P. gingivalis,* a prominent pathogen of periodontitis [40, 41]. Nanoparticle tracking analysis of the isolated samples detected >10^9^ particles (/ml) stained with a lipophilic dye (Supplementary Fig. 1A). Transmission electron microscopy (TEM) revealed the presence of spherical particles in the collected sample (Supplementary Fig. 1C), and no prophage genes including those related to lipid enveloped virus was identified in this strain [42], indicating that BEV is dominant nanoparticles in the bacterial cell cultures. To understand the composition of the DNA within these vesicles, shotgun sequencing for the bulk BEV samples was performed (Fig. 1A), after treatment with DNase I to remove any external DNA (eDNA). The sequence reads from these DNA were widely mapped to the original bacterial genome; however, several genomic regions were over-detected (Fig. 1B), suggesting selective DNA packaging within the BEV population.

To check the overrepresentation of specific genomic regions at a higher resolution, WGA of these particles was performed using a droplet-based method [25]. The samples were mixed with agarose such that ≈40% of the droplets contained a single nanoparticle (Table 1, Supplementary Fig. 2, denoted as *R_t_*). This process harbored 4.6% of the droplets contained amplified DNA (stained with SYBR-Green-I, Table 1, Supplementary Fig. 2, represented as *Ro*). Given that nearly 20% of the total nanoparticles were lipid-stained (Table 1, Supplementary Fig.1, 2, presented as *R_b_*), we could estimate that ≈60% of lipid-stained nanoparticles (BEVs) harbored DNA (Table 1). This substantial population underscores the prevalent DNA packaging process within the BEVs of *P. gingivalis*, substantially surpassing the value estimated by SYBR-Green-I (a dye for nucleic acids) directly on BEVs like a conventional method (0.44 ± 0.32%) of positively stained BEVs in NTA (Supplementary Fig. 1). This discrepancy between two methods is likely attribute to the low-permeability of the dye into the bacterial cytoplasmic space or the presence of too short DNA fragments to be detected by this technique, given fluorescence staining sensitivity is contingent on DNA length [43].

**Table 1:** Estimated proportion of BEVs containing DNA in the analyzed population. The ratio of droplets to particles with DNA (*R_o_*). The theoretical ratio of droplets to total particles (*R_t_*) was controlled to a fixed value in our analysis. *R_o_* and *R_vb_* were estimated by NTA and taxonomic annotation, respectively. The proportion of BEVs with DNA, *r,* was calculated based on those parameters. For calculation details, see Supplementary Methods and Supplementary Fig. 2.

	*P. gingivalis*	Biofilm
Particles with DNA / Droplets (*R_o_*)	0.046	0.035
Total particles / Droplets (*R_t_*)	0.36	0.36
Viruses / BEVs with DNA (*R_vb_*)	0	3.97
Total BEVs / Total nanoparticles (*R_b_*)	0.21	0.053
BEVs with DNA / Total BEVs (*r*)	0.62	0.35

To determine the DNA content of individual BEV particles, whole-genome sequencing of DNA amplified by MDA was performed. Of the 96 droplets, most sequence reads were mapped to the *P. gingivalis* genome in 93 droplets (Supplementary Fig. 3), and wide regions of the host chromosome were detected in at least one droplet, similar to the profile in bulk-BEV sequencing (Fig. 1B). Although there were variations in the detected genomic regions within the BEV-population from *P. gingivalis* (Fig. 1B), 10–30 kb of genomic regions were detected in ≈70% (65/96) of droplets (Fig. 1C). By calculating the continuous genomic regions mapped by the sequence reads within each droplet, we found that in nearly 70% of droplets (68/96), the majority (>80% in length) of the detected genomic regions originated from the single longest genomic region (Supplementary Fig. 4). This suggests that most of the DNA fragments in each BEV derived from one single genomic locus. Some droplets containing DNA from the multiple chromosomal loci (Supplementary Fig. 4), and such droplet would package a BEV possibly containing the DNA fragments from substantially different genomic loci or multiple BEVs with DNA from different genomic loci. However, the distribution of the total detected regions and the longest continuous detected regions exhibited quite similar trend (Fig. 1C), and such BEVs or droplets were minor in the analyzed population.

Although the regions mapped by read sequences from *P. gingivalis*-BEVs were not uniformly distributed among the 96 droplets (Fig. 1B), we found a few genomic regions that were significantly over-detected among the BEV population (binominal test*, P* < 0.01, see Supplemental Methods) (Fig. 1B). Common enriched regions existed between bulk-BEV sequencing and droplet sequencing of BEVs from *P. gingivalis* (Fig. 1B), yet numerous frequently mapped regions in bulk-BEV sequencing failed classification as enriched based on detected droplet criteria. A deviation between the two approaches was also observed in the prevalence of each protein CDSs. The mapped sequence reads (FPKM) in bulk-BEV-sequencing exhibited longer-tailed abundance distribution of 1873 CDSs compared to the detected droplets in NP-DS (Fig. 1D). Consequently, the bulk-BEV-sequencing identified approximately seven times more enriched CDSs (i.e. outliers in the distribution; Supplementary Data 1) than NP-DS (Fig. 1D). This deviation occurred because mapped sequence reads in bulk-BEV sequencing responded significantly to GC content of each genomic locus (Supplementary Fig. 5), as reported in previous studies [44, 45], whereas GC content minimally affected detected droplet counts (Supplementary Fig. 5).

We validated enrichment of specific genomic regions in BEVs using a simpler approach; quantitative PCR (qPCR) analysis. We targeted two regions: one with significantly enrichment (magenta in Fig. 1B, bottom) and another with zero droplet detection (gray in Fig. 1B, bottom). Results showed high detection levels for the enriched region (0.06 ± 0.03 copies/BEV particle) (Fig. 1F), but the other target site showed minimal detection using qPCR (0.002 ± 0.003 copies/BEV particle) (Fig. 1F), aligning with NP-DS results.

We applied the qPCR analysis to extracellular DNA (eDNA) from the *P. gingivalis* culture supernatant, finding that the DNA purified from the eDNA fraction contained fragments of DNA from both BEV enriched and nonenriched genomic regions at a comparable level (Supplementary Fig. 6), and this tendency was distinct from that observed in the extracted DNA from equivalent amount of BEV fraction (Supplementary Fig. 6). This result indicates that the enrichment of specific DNA was only observed in the BEV fraction in our experiments, and thus most of the DNA observed in our analysis was derived from BEVs but not eDNA remnants.

### DNA profiles in BEVs were susceptible to the biogenesis mechanism

Although membrane blebbing and explosive lysis followed by recircularization are reportedly major mechanisms of BEV formation [14, 46, 47], the mechanism of DNA packaging into BEVs remains debatable. To address this issue, the selective packaging of specific genomic loci into BEVs was compared between these two mechanisms. First, cells were treated with artepillin-C, which promotes BEV formation via membrane blebbing [48] (Fig. 1E). The copy numbers of the DNA fragments from the target genomic regions (Fig. 1B, bottom; gray and magenta) were quantified using qPCR. Despite an increase in BEV production (Supplementary Fig. 7B), the same genomic regions were detected at levels comparable to those of the untreated controls (Fig. 1F), indicating that artepillin-C treatment did not significantly alter the DNA profile of BEVs.

When cells were treated with petrocelinic acid (PA), BEV biogenesis was induced by explosive cell lysis or bubbling cell death [49] (Fig. 1E), where the genomic regions frequently detected in naturally occurring BEVs (i.e. those produced during normal cell growth) were barely detectable in PA-induced BEVs (Fig. 1G). This suggests that membrane blebbing rather than cell lysis, is responsible for the enrichment of specific DNA fragments under normal growth conditions.

PA-induced cell lysis harbored both BEV enriched and nonenriched genomic regions in eDNA fraction at a comparable or higher level in a BEV fraction (Supplementary Fig. 6), and thus this process contribute to the increase in eDNA amount similar to a previous study [46]. However, both genomic regions were barely detected in PA-induced BEVs (Supplementary Fig. 6) and total DNA was ~10-fold lower than that extracted from naturally occurring BEVs (Supplementary Fig. 8), suggesting that cell lysis and the accompanying increase in eDNA do not contribute as effectively to the selective DNA packaging into BEVs like blebbing case.

### Enrichment of functional genes in BEVs from *P. gingivalis*

**Figure 2:**
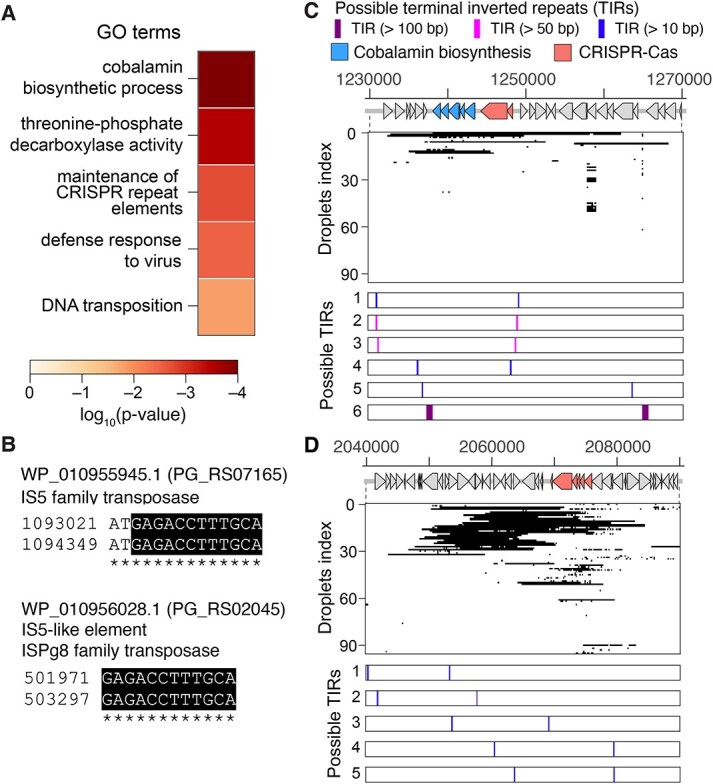
Functional enrichment of genes in *P. gingivalis* BEVs. (A) Enriched gene categories in CDSs detected in BEVs of *P. gingivalis*. Frequently detected CDSs in BEVs were statistically extracted (hypergeometric test, *P* < 0.05, see supplementary methods). Gene ontology terms (GO terms) were used for the classification of the frequently detected CDSs. Heatmap indicates the statistical significance level of enrichment. (B) The identified possible TIRs adjacent to the overrepresented IS elements in the *P. gingivalis* BEVs. Asterisks indicate the aligned nucleotide pairs between left-arm and right-arm of the inverted repeats. Shaded regions are the conserved nucleotide sequences between those two transposases. (C), (D) Clustered functional genes located on the enriched genomic regions in *P. gingivalis* BEVs. Detection profiles in 96 droplets where (C) cobalamin biosynthetic genes and type VI-B2 CRISPR-Cas gene cluster or (C) type III-B CRISPR-Cas gene cluster located. In each row, the positions on the genome where the sequence reads were mapped in each droplet are filled. In the bottom, possible TIRs located around each region were plotted. Each TIR pair was colored by its length.

We investigated the functional implications of DNA enrichment in *P. gingivalis* BEVs. To explore the genes loaded into a large fraction of the BEV population, the over-detected CDSs were statistically extracted among 96 droplets in NP-DS, revealing 39 significantly over-represented CDSs among the 1873 present in the genome (Supplementary Table 1). A homology search of the enriched CDSs in BEVs against the UniProtKB/Swiss-Prot database [50] enabled GO terms categorization of the statistically screened genes (Fig. 2A and Supplementary Table 2). Five categories among 497 showed statistical enrichment (hypergeometric test, *P* < 0.05, Supplementary Methods). “DNA transposition (GO:0006313)” emerged significant, indicating the predominance of genome-arrangement functions in the BEV-derived DNA. ~20% of the enriched CDSs were related to genome arrangements such as transposase, site-specific integrase, and CRISPR-Cas system (9/39). The transposase and integrase are flanked by the terminal inverted repeats (TIRs) (Supplementary Data 2) and possess the typical excision signatures in their adjacent region (Fig. 2B). ≈15% (13/96) of droplets contained IS*5* family transposases (WP_010956028.1 and WP_010955945.1) with the conserved regions in TIR (Fig. 2B), suggesting that specific transposases with the specific excision signatures are associated with BEV biogenesis and DNA-packaging.

The CRISPR category, linked to “Maintenance of CRISPR repeat elements (GO:0004803)” and “defense response to virus (GO:0051607)”, was particularly highlighted by the packaging of an entire CRISPR-Cas gene cluster within an enriched genomic region (Fig. 2D), quite similar tendency as DNA in BEVs from the marine microbiota [24]. The genes grouped into “Cobalamin biosynthetic process (GO: 0009236)”, another enriched functional category (Fig. 2C), is clustered at a specific locus (Fig. 2C). This cobalamin biosynthesis cluster is also adjacent to the type VI-B2 CRISPR-Cas gene clusters (Fig. 2C), indicating the strong relationship between the CRISPR-Cas gene clusters and selective packaging into BEVs in this strain.

Bulk-BEV sequencing produced the bias derived from the GC content and also harbored approximately triple the screened categories compared NP-DS in GO term analysis, spanning metabolic, stress response, and enzymatic categories (Supplementary Table 3). Nevertheless, identical or similar GO terms emerged, including “cobalamin biosynthetic process (GO: 0009236)” and “threonine-phosphate decarboxylase activity (GO:0048472)” (Supplementary Table 3), demonstrating methodological consistency.

We characterized the chromosomal location and excision signatures of the overrepresented gene clusters. The packaging of DNA fragments excised by Xer recombinase at *dif* site into BEVs was previously reported [51], but any concordance of the *dif site* and the enriched genomic loci in *P. gingivalis* was found (Supplementary Fig. 9). Then, we focused on TIRs, more common excision signatures, as in the case of, and identified the possible candidates of TIRs around the enriched genomic regions (Fig. 2C and D, Supplementary Fig. 10, and Supplementary Table 4). Those identified TIRs are usually ≈14 bp, yet we found the long tandem inverted repeats flanking cobalamin biosynthesis gene cluster (≈600 and ≈50 bp, Supplementary Fig. 10 and Fig. 2C and D), suggesting the high probability of excision in this region, given the high excision frequency of the long inverted repeats [52]. TIR-flanked regions on the *P. gingivalis* genome were generally more prevalent than other regions in the BEVs (Supplementary Fig. 11), implying correlation between the presence of TIRs and the selective packaging in this bacterium genome. However, most of the identified TIRs adjacent to the targeted gene clusters were not found in the other loci and unique on the *P. gingivalis* genome (Supplementary Data 3), which also implies that specific excision signature relates to the selective packaging.

### Enrichment of gene clusters that are possible hotspots of horizontal transmission

**Figure 3:**
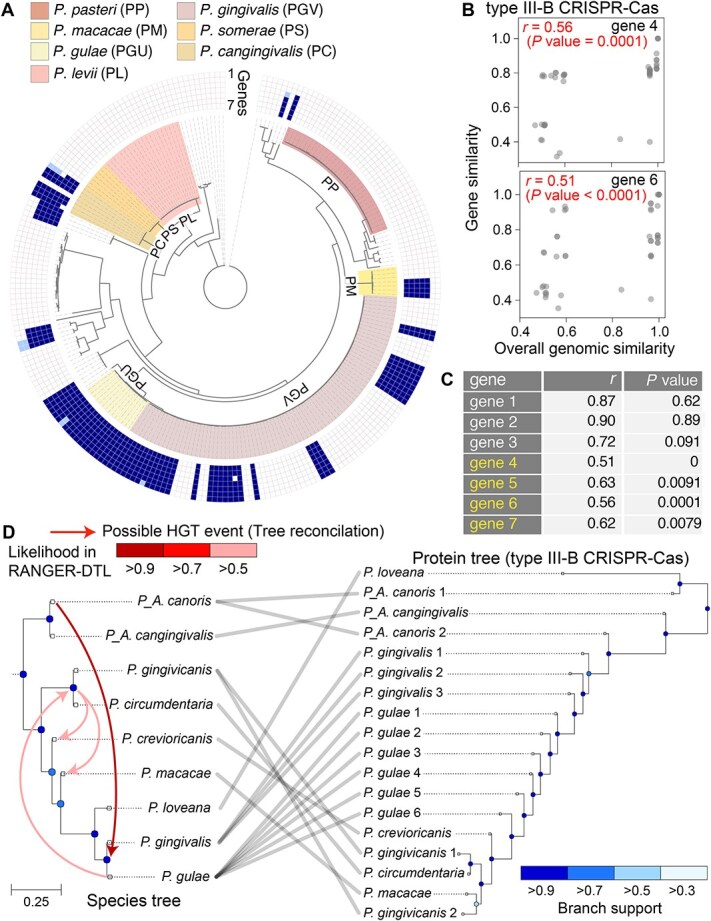
Prevalence and possible evolutionary history of the gene-cluster enriched in the BEVs in *Porphyromonas* group. (A) The prevalence of the seven genes in the CRISPR-Cas cluster among 244 genomes from the *Porphyromonas* group is displayed on the phylogenetic (species) tree tips. The presence of the genes in each genome is displayed as a heatmap from gene 1 to 7 (the number corresponds to the gene arrows in Fig. 2). The genes existing on the genome as a cluster are indicated with darker shading. If the gene is present but located on the distant locus from the gene cluster, it is indicated with lighter shading. The major species group is shaded by colors in the phylogenetic tree. (B) Discordance between the overall similarity of the genome and the similarity of the genes in the CRISPR-Cas cluster across the *Porphyromonas* species. Pearson’s correlation (*r*) between two similarity scores is displayed. The *P*-value indicates the probability that the randomly selected gene similarity score exhibited a lower correlation than the observed value in the permutation test (see supplementary methods). (C) The correlation between the overall similarity of the genome and the similarity of the genes in all analyzed members. The numbers correspond to those in Fig. 2. The statistical significance levels of correlation values are displayed as *P* values. (D) Comparison of the concatenated gene-cluster tree of CRISPR-Cas cluster and the corresponding species tree. The blue dots on the internal nodes of the trees show the branch support values. The red arrows indicate possible the horizontal gene transfer (HGT) events predicted by the tree reconciliation approach by RANGER-DTL. The color density indicates the likelihood that the corresponding HGT was observed in RANGER-DTL analysis (see supplementary methods).

DNA fragments in BEVs are potentially transferred to other bacteria [16–18], suggesting that enriched regions identified in our analysis could be horizontally transmitted among this bacterial group during evolution. We investigated the possibility of HGTs for two representative gene clusters: cobalamin biosynthesis and type III-B CRISPR-Cas gene clusters (Fig. 2C and D). Analysis of 244 high-quality genomes in the *Porphyromonas* genus revealed the prevalence of both clusters across diverse *Porphyromonas* species (Fig. 3A and Supplementary Fig. 12), yet type III-B CRISPR-Cas cluster appeared only in specific strains within each species, including *P. gingivalis* (Fig. 3A), indicating limitations of vertical transmission mechanism in explaining its distribution among *Porphyromonas*.

We tested the possibility of horizontal transmission of the CRISPR-Cas cluster among *Porphyromonas* species using a comparative genomics approach. Horizontally transferred genes often show evolutionary history differing from species-level phylogenies, manifesting as phylogenetic distances discrepancies between genes and species particularly in comparison to other genes [53, 54]. Comparison of the similarity of CRISPR-Cas genes with overall genomic similarities within the *Porphyromonas* group revealed significant deviations between those two parameters in four of the seven cases (Fig. 3B and C), suggesting that HGT events contributed to the dissemination of this gene cluster. In contrast, cobalamin biosynthesis genes showed no such phylogenetic discrepancies (Supplementary Fig. 12), suggesting primarily vertical transmission.

To further corroborate the possibility of horizontal transmission, two phylogenetic trees were directly compared, one based on whole-genome sequences (general marker genes) and the other based on the five genes in the CRISPR-Cas cluster that are widely present in *Porphyromonas*, resulting in considerable discrepancies between those two trees (Fig. 3D). Tree reconciliation analysis identified four potential species-level HGT events within *Porphyromonas* (Fig. 3D). These findings support horizontal transmission of CRISPR-Cas cluster among *Porphyromonas* species, emphasizing the potential role of BEVs in the evolutionary dynamics of this gene cluster.

### DNA profiling of nanoparticles from human oral biofilm

**Figure 4:**
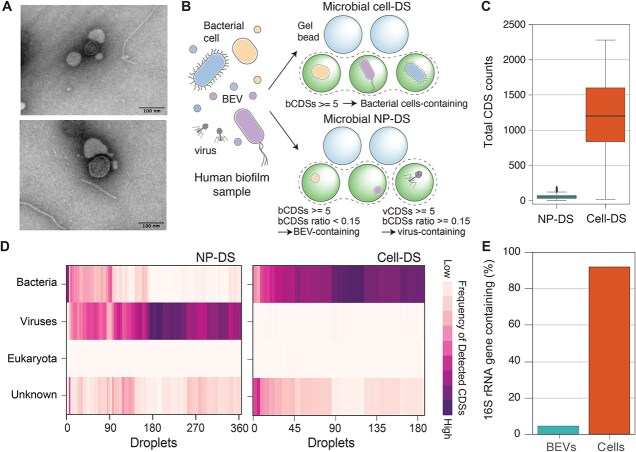
Distinct characteristics of DNA in BEVs compared to bacterial cells. (A) The typical transmission electron microscope (TEM) micrographs of BEVs isolated from the dental plaque of periodontal patients. (B) Schematic of microbial cell-DS and NP-DS analysis of the dental plaque biofilm samples from periodontal patients. The separated nanoparticles and bacterial cells in dental biofilms were encapsulated into droplets, and the internal DNA fragments were amplified (see materials and methods). In NP-DS, the positive amplification droplets (enclosed by dashed lines) were classified by the number of detected bCDSs and vCDSs as BEV-containing, viral DNA-containing, or both. (C) The total length of detected CDSs in NP-DS and cell-DS analysis. The results of all positive droplets from the biofilm samples were analyzed and are shown as boxplots. Medians and outliers are shown as bold lines and circles, respectively. (D) Kingdom-level classification of detected CDSs in NP-DS and cell-DS. In each droplet, the length of CDSs assigned to bacteria, viruses, eukaryotes, or unknown (i.e. not assigned to any taxon in the database) was normalized by the total length of detected CDSs and is shown as a heatmap. The droplets where less than five CDSs were detected were eliminated. (E) The percentage of BEV- or bacterial cell-containing droplets in which 16S rRNA sequence was detected. The DNA sample is a mixture of BEVs collected from the biofilms of three patients.

We attempted to characterize DNA content in BEVs produced in the dental plaque biofilm of periodontal patients, the natural habitat of oral pathogenic bacteria. Transmission electron micrographs of the isolated samples revealed spherical particles with diameters in the range of ≈100–200 nm (Fig. 4A). NTA also detected particles stained with lipophilic and double-stranded DNA dyes (Supplementary Fig. 13A), with DNA-positive particles exceeding lipid-stained particles (Supplementary Fig. 13B), suggesting presence of nanoparticles with DNA that do not consist of a lipid layer, such as viruses. However, the amount of extracted DNA was below the minimum amount required for conventional metagenome sequencing (<1 ng per sample).

Droplet sequencing was successful for collected BEVs with low DNA quantities. Following the protocol for *P. gingivalis*, the DNA-positive gel beads in NP-DS and lipid-stained nanoparticles were quantified (Supplementary Fig. 2), and the fraction of nanoparticles containing DNA was estimated as ≈10% (Supplementary Fig. 2B and C), matching the number of DNA positive particles in NTA (Supplementary Fig. 13B). Next 384 positive droplets were sequenced (Fig. 4B). The sequence reads from each droplet were computationally assembled, divided into CDS units, and searched against the nr database (see Materials and Methods). This method detected an average of 52 ± 29 CDSs per droplet (Fig. 4C), predominantly virus (bacteriophage)-derived (vCDS). TEM analysis revealed the presence of particles with the morphology of tailed bacteriophages (Supplementary Fig. 14A), and all the vCDSs were assigned to *Caudovirales* (Supplementary Fig. 14B). Thus, the origin of most viral DNA would be phage particles. However, we also observed several droplets containing both bCDSs and vCDSs; the origin of the viral DNA in such cases will be discussed later. Approximately 20% of droplets (73/384) contained ≥5 bacterial CDSs (bCDSs) (Fig. 4D), and bCDSs / (vCDSs + bCDSs) ratio in those droplets >0.15 (Supplementary Fig. 15) and regarded as BEV-containing based on a previous comprehensive study of auxiliary metabolic genes in viral genomes [55]. However, CDSs annotated as host (human)-derived were barely detected (Fig. 4C, Eukaryote). Considering the fraction of virus-containing droplets in NP-DS, the ratio of BEVs with DNA to the total BEVs was estimated as ≈30% (Table 1, Supplementary Fig. 2, presented as *r*). Thus, a significant fraction of BEVs contained DNA in the oral biofilm.

The DNA content of a BEVs was compared to that of a bacterial cell by droplet sequencing of bacterial cells (cell-DS) isolated from the same dental plaque samples (Fig. 4B). In cell-DS, an averagely of 1204 ± 542 CDSs and 790 ± 490 kb of CDS region was detected (Fig. 4C and Supplementary Fig. 16A), and full-length 16S rRNA sequences were observed in >90% of the droplets (Fig. 4E). In contrast, the CDS regions detected in most BEV-containing droplets were ≈9 kb in each droplet (8.6 ± 6.5 kb, Supplementary Fig. 16B), corresponding to ≈1% of those detected in cell-DS. In addition, full-length 16S rRNA sequences were detected in 4% (3/68) of BEV-containing droplets (Fig. 4E).

### Enrichment of specific genomic regions in BEVs from the oral cavity

**Figure 5:**
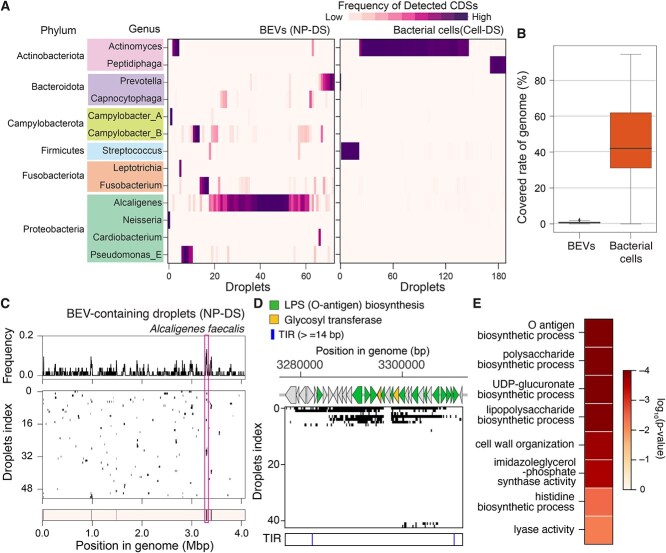
Enriched DNA sequences in BEV-containing droplets. (A) Genus-level taxonomic classification of bCDSs detected in BEV-containing droplets in NP-DS and bacterial cell-containing droplets in cell-DS. The length of the CDSs assigned to each bacterial genus group was normalized to the total detected bCDSs and are shown as a heatmap. We only display the genera whose maximum frequency of detected CDSs in a single droplet is more than 0.4. (B) The percentage of the genomic region covered by the sequence reads in a BEV- or bacterial cell-containing droplet. For each droplet, sequence reads are aligned to the assembled genome of the MFT. Medians and outliers are shown as bold lines and circles in box plots, respectively (C) genomic regions in *A. faecalis* (GCF_002443155.1) mapped by sequence reads in BEV-containing droplets. The frequency of droplets, including each genomic region (top). In each row, the positions on the genome where the sequence reads are mapped in each droplet are filled with black (middle). The genomic regions that were significantly enriched in BEV-containing droplets are filled with red (bottom). We analyzed the sequence data of 53 BEV-containing droplets whose MFT was determined as *A. faecalis* (GCF_002443155.1). (D) Detection profiles of the lipopolysaccharide (LPS) biosynthesis gene cluster (encircled by magenta in panel C) in 53 droplets. The color of each gene arrow corresponds to the functional group. (E) Screened functional categories (GO terms) that were significantly overrepresented in the enriched genomic region of BEVs from *A. faecalis*. Similar to Fig. 2, we statistically extracted the GO terms (hypergeometric test, *P* < 0.05, see supplementary methods). The statistical significance level is shown as a heatmap.

Taxonomic annotation of the detected bCDSs in each BEV-containing droplet through homology search against the GTDB (Supplementary Fig.17) revealed dominance of bCDSs from a single phylum, family, or genus-group (~70% –90% of purity, Fig. 5A and Supplementary Fig. 18), suggesting that the most frequently detected bacterial taxon (MFT) for each droplet reflected the BEV origin. Mapping of the sequence reads to the assembled genome of MFT (Supplementary Fig. 17) revealed that BEV-containing droplets covered 0.5%–1% of the genomic regions, whereas droplets in cell-DS reasonably covered 40%–60% of the genomic regions (Fig. 5B).

BEVs from dental plaque biofilms also exhibited enrichment of specific genomic regions, similar to the pure culture system. Analysis of droplets with an MFT derived from *A. faecalis* (GCF_002443155.1), the predominant taxon in BEV-containing droplets (Fig. 5C), revealed the presence of 5–30 kb of the continuous genomic regions in ≈70% of those droplets (Supplementary Fig. 19), which were distributed across a wide range of host chromosomal regions (Fig. 5C). However, some regions were commonly detected among the droplets (Fig. 5C, binomial test *P* < 0.01), including a genomic locus consisting of many lipopolysaccharide (LPS) biosynthesis genes, which has a possible TIR in its adjacent region (Fig. 5D and Supplementary Table 5). A statistical screening of enriched functional categories also identified GO terms associated with bacterial antigen (LPS) biosynthesis, such as “O antigen biosynthetic process (GO:0009243)” and “LPS biosynthetic process (GO:0009103)” (Fig. 5E and Supplementary Table 6). O-antigen biosynthesis genes appeared in ≈14% of *A. faecalis*-derived BEVs (≈10% of all analyzed DNA-containing BEVs in the oral biofilm), suggesting the prevalence of these genes in the biofilm BEVs.

### Origins of BEV-derived DNA in dental plaque

Taxonomic profiles of bCDSs detected in the NP-DS were markedly distinct from those detected in the cell-DS. At the phylum level, abundant groups in BEV-containing droplets, such as *Proteobacteria*, were barely detected in the cell-DS (Fig. 5A); thus, most of the DNA sequences detected in BEVs were derived from bacterial species with very low abundance in dental plaque biofilms. Indeed, *Actinomyces, Peptidiphaga,* and *Streptococcus* were the most frequently detected taxa and MFTs in ~90% of droplets in the cell-DS, whereas these CDSs were barely detected in the NP-DS (only in six droplets) (Fig. 5A). In contrast, CDSs from *Alcaligenes* (*Proteobacteria*), the most abundant genus detected in BEV-derived DNA, were quite low in frequency in the cell-DS (Fig. 4A). In addition to *Alcaligenes*, we also found the bCDSs from several minor bacterial genera in the cell-DS, such as *Prevotella, Capnocytophaga, Campylobacter, Leptotrichia,* and *Pseudomonas_E*, in the NP-DS, although some of these (e.g. *Prevotella*) were previously reported to be prevalent in periodontal oral biofilms [56]*.* These results suggest that the BEV-derived DNA detected using our method could reveal the distribution of functional genes among particles and highlight the taxonomically minor but active producer strains of BEVs in the microbiome.

## Discussion

The present study revealed that ≈40% of the BEV population of *P. gingivalis* and ≈ 20% of the BEVs from the oral biofilm (corresponding to ≈70% of the BEVs from *A. faecalis*) contained 10–30 kb or 5–30 kb fragments respectively. These findings indicate that a considerable fraction of BEVs from the oral microbiota contain DNA fragments with multiple genes, with certain functional gene clusters being prevalent across the BEV population (Fig. 2, and Fig. 5). NP-DS analysis revealed gene enrichment in *P. gingivalis* BEVs associated with functions more specific than those identified by bulk sequencing (Fig. 1). Bulk sequencing tended to capture a broad array of miscellaneous gene sets (Supplementary Data 1) and was susceptible to amplification bias during library preparation (Supplementary Fig. 5). It is possible that DNA amplification within individual droplets, combined with count-based statistical analysis, mitigates these biases, leading to a precise identification of functionally enriched genes within BEVs. Furthermore, the high sensitivity of droplet-based approach for detecting DNA-containing BEVs (Supplementary Fig. 2) has the potential to further clarify the ubiquity of BEVs as DNA carriers in microbial communities in diverse environments.

The enrichment profiles, the prevalence of DNA content, and their unique evolutionary history strongly suggest the potential ecological impacts of DNA cargoes on the microbial community via HGT. Enrichment of a specific group of metabolic or virulence-associated genes was found in both bacterial cases (Fig. 2A and Fig. 5E), similar to other mobile genetic elements (e.g. plasmids and integrative conjugative elements) [57, 58].Our phylogenetic analysis revealed discordance between the evolutionary histories of species and genes in the BEV-enriched gene cluster (Fig. 3), suggesting that BEVs contain HGT hotspots. Clustered gene sets were estimated to be present in 3%–12% of BEVs of *P. gingivalis*, which was a 10–100-fold greater than those reported in the other bacteria [24]. Although one possible reason for this deviation would be the difference in bacterial species and growth condition, our results in *P. gingivalis* using traditional DNA dye staining techniques also detected <1% of DNA positive BEVs, and thus it is possible that DNA-packaging BEVs are far more prevalent than those estimated by the direct staining.

The “O-antigen biosynthesis genes” were highly prevalent in the oral plaque biofilm-derived BEVs. These gene sets are generally clustered on the genome of Gram-negative bacteria [59, 60], and the high sequence diversity and unique GC content of this genomic region suggest that this gene cluster is a frequent site for HGT [61, 62]. Given that divergence in O-antigens can potentially impact host–bacterial interactions [63], it is possible that the enriched genes in BEVs modulate the pathogenicity of the oral biofilm via HGT.

*P. gingivalis* BEVs showed enrichment of genes associated with DNA integration, including DNA transposition, and CRISPR-Cas system, which are also consistent with the prevalence of mobile genetic elements (MGEs) in BEVs from marine environment [13]. Insertion sequences (IS) are believed to facilitate genome rearrangements in this species group [64]. Furthermore, comparative genomic analyses revealed discordance between species and gene similarities in the CRISPR-Cas cluster among this bacterial group (Fig. 3), strongly suggesting a critical role for HGTs via BEVs. CRISPR spacer sequences are highly homologous to IS regions in the *P. gingivalis* genome, potentially preventing IS transposition and recombination [64]. In addition, targets for some spacer sequences correspond to prophages spread among the *Porphyromonas* group [42]. The enrichment of CRISPR-Cas elements and the traces of lateral transfers suggest that BEVs in this bacterium play an important role in the interspecies recombination of IS and in defense against prophages through HGT. Although the frequency and extent of gene transfer among bacteria requires further investigation, our study lays the groundwork for identifying transferable gene candidates via BEVs and their potential effects on microbial community.

The overrepresentation of CRISPR-Cas elements and IS elements in *P. gingivalis* BEVs also provides significant clues for elucidating the mechanisms of the selective packaging. The BEV DNA is rich in IS elements with typical TIRs (Fig. 2B), suggesting that these DNA regions tend to be excised and loaded into BEVs via certain mechanisms. The potential of Cas1-containing elements (so called Casposon) as mobile genetic elements has been posited from the aspects of presence of TIRs, the integrase activity of Cas protein, and its potential role in the evolution of CRISPR-Cas [65–67]. Although the identified enriched genomic regions in BEVs in the current study (Fig. 2) and previous one [13] is the typical CRISPR-Cas cluster rather than Casposons, our genomic analysis also identified the TIRs possibly works as excision sites (Fig. 2), and the trace of the horizontal transmission in the type III CRISPR-Cas element among *Porphyromonas* (Fig. 3), suggesting that its potential as a MGE in BEVs via the excision from the chromosome and subsequent packaging into BEVs. In contrast, the excision by Xer family recombinase at *dif* sequences, one of the widely observed recombination system in bacteria [68], would not be the mechanism of the selective DNA packaging in the targeted bacteria given the no concordance of the *dif* site and the overrepresented genomic loci in both bacteria (Supplementary Fig. 9).

Our findings highlight the enrichment of specific genomic loci in BEVs and reveal the requirements of certain biogenesis routes for this enrichment. A biogenesis route for DNA-containing BEVs that aligns with membrane blebbing was previously proposed [69], where the peptidoglycan layer is locally and transiently weakened by autolysins, facilitating the translocation of cytoplasmic content, including chromosomal DNA—a process observed in *P. gingivalis* BEVs [70]. Moreover, outer-inner membrane vesicle formation through blebbing proposed as the major mechanisms of DNA packaging across Gram-negative bacteria [22, 23, 71], supporting the prevalence of specific mechanisms for DNA enrichment across bacterial species. Excision of IS elements can be also influenced by environmental stressors or specific genetic factors [72, 73], which may alter the frequency of their incorporation into BEVs depending on the type of stress [74]. Another mechanism is that BEVs take up free eDNA through a process similar to natural transformation [75]. However, given the biased gene profile derived from BEVs, eDNA contamination was not a major factor (Supplementary Fig. 6). These results suggest that the DNA cargo in *P. gingivalis* BEVs is not simply a byproduct of cell death (lysis) but rather a result of specific biological mechanisms.

Taxonomic classification in NP-DS underscore the rarity of 16S rRNA sequences within BEVs (Fig. 4E), indicating that shotgun-based approach provided a more comprehensive view of the taxonomic composition of BEV-derived DNA than 16S rRNA-based approach. Our analysis revealed substantial taxonomic differences between the profiles obtained from cell-DS and NP-DS (Fig. 5A). This discrepancy suggests that minor members produce BEVs or package DNA into BEVs more actively than major bacterial groups in human dental plaques. NP-DS identified DNA from several other pathogens (e.g. *Capnocytophaga* and *Leptotrichia*), which were minor bacterial groups in our cell-DS data (Fig. 4A); however, some of these were prominent pathogens in periodontitis [76]. Given the highly organized structure of the oral biofilm [77], it is also possible that bacterial species that tend to form large aggregates or become trapped in biofilms are less frequently detected by droplet sequencing, whereas BEVs from these bacterial taxa are more easily released into the environment because of their small size. Assuming that DNA encapsulation into BEV reflects the physiological status of the original cells, we can assume that these enriched DNA regions may serve as biomarkers to assess the physiology of such pathogens. Therefore, the unique characteristics of BEV-derived DNA imply its potential applicability in biopsy for diagnosis.

Our analysis also detected a large amount of phage-derived DNA in NP-DS. Although the origin of these viral DNA fragments would be mostly phages themselves, ~20% of the droplets contained both bacterial and viral CDSs (i.e. containing 15% or more bCDS of vCDS). Although such mixed particles would partly result from the aggregations in the purification procedure, incorporation of viral fragments into BEVs by biological processes such as phage induction and infection in bacterial cells [21, 46] or phage infection to BEVs [78] would be possible. Further experimental investigation is necessary to understand the coexistence of bacterial and phage DNA, yet droplet sequencing could be a potential tool to explore phage-BEV interactions in nature.

## Supplementary Material

Supplemental_materials_20250825plain_wraf193

Supplemental_materials_20250825plain_wraf193_1

Supplementary_Data_wraf193

## Data Availability

All sequence raw data used in this study were deposited in the DNA Data Bank of Japan (DDBJ) with the accession ID PRJDB17260 and PRJDB17266. The intermediate analysis data were also deposited in *Zenodo* (doi:10.5281/zenodo.16662605). Codes for data analysis can be found in the GitHub repository: https://github.com/sotarotakano/DropletSequenceAnnotator_2025.
