# Supplementary material for: Enrichment of horizontally transferred gene clusters in bacterial extracellular vesicles via non lytic mechanisms
Source: ISME J. 2025 Aug 29;19(1):wraf193. doi: 10.1093/ismejo/wraf193 (PMC12477599; doi:10.1093/ismejo/wraf193)
Supplement: Supplemental_materials_20250825plain_wraf193 [file supplemental_materials_20250825plain_wraf193.pdf]

Supplementary Information

Enrichment of Horizontally Transferred Gene Clusters in Bacterial Extracellular Vesicles via Non-Lytic Mechanisms

Sotaro Takano[1,2], Satoshi Takenawa[1], Naradasu Divya[1], Kangmin Yan[1], XinXin Wen[1], Tomoko Maehara[1], Nobuhiko Nomura[3,4], Nozomu Obana[4,5], Masanori Toyofuku[3,4], Michihiko Usui[6], Wataru Ariyoshi[7], Akihiro Okamoto[1,3,8,9*]

1 Research Center for Macromolecules and Biomaterials, National Institute for Materials Science, 1-1 Namiki, Tsukuba, Japan, 305-0044
2 Integrated Bioresource Information Division, BioResource Research Center, RIKEN, 3-1-1 Koyadai, Tsukuba, Japan, 305-0074
3 Graduate School of Life and Environmental Sciences, University of Tsukuba, 1-1-1 Tennodai, Tsukuba, Japan, 305-8577
4 Microbiology Research Center for Sustainability, University of Tsukuba, 1-1-1 Tennodai, Tsukuba, Japan, 305-8577
5 Transborder Medical Research Center, Faculty of Medicine, University of Tsukuba, University of Tsukuba, 1-1-1 Tennodai, Tsukuba, Japan, 305-8577
6 Division of Periodontology, Department of Oral Function, Kyushu Dental University, 2 Chome-6-1 Manazuru, Kokurakita Ward, Kitakyushu, Fukuoka, Japan, 803-8580
7 Division of Infection and Molecular Biology, Department of Health Promotion, Kyushu Dental University, 2 Chome-6-1 Manazuru, Kokurakita Ward, Kitakyushu, Fukuoka, Japan, 803-8580
8 Graduate School of Chemical Sciences and Engineering, Hokkaido University, North 13 West 8, Kita-ku, Sapporo, Japan, 060-8628
9 Research Center for Autonomous Systems Materialogy, Institute of Innovative Research, Tokyo Institute of Technology, 4259 Nagatsuta-cho, Midori-ku, Yokohama, Kanagawa, Japan, 226-8501

*Akihiro Okamoto
Research Center for Macromolecules and Biomaterials, National Institute of Materials Science (NIMS)
1-1 Namiki, Tsukuba, Ibaraki 305-0044, Japan.
Email: OKAMOTO.Akihiro@nims.go.jp

This PDF file includes:

Supplementary Methods
Supplementary Figures 1 to 20
Supplementary Tables 1 to 7
Legends for Supplementary Datasets 1 to 5 (Separate .xlsx file)
SI References

Supplementary Methods

Droplet genome sequencing.

Droplet genome sequencing was performed by bitBiome (Japan) as previously described [1]. Briefly, in the cell-DS, the cell suspensions from biofilm samples were mixed with 1.5% agarose solutions in Dulbecco's PBS (DPBS; Thermo Fisher Scientific) so that the expected percentage of positive droplets was ≈ 40% (i.e. cells/droplets ratio become ≈ 0.5) based on the previous study [2]. In the case of nanoparticle genome sequencing, the suspensions of nanoparticles were mixed with the agarose solutions so that the expected percentage of positive droplets was less than 30%. Bacterial cells or nanoparticles were encapsulated in droplets using fabricated microfluidic droplet generators [3] and further used for in-bead bacterial genome amplification sequencing. Collected droplets were incubated on ice for 15 min for solidification. The solidified gel droplets were broken with 1H,1H,2H,2H-perfluoro-1-octanol (Sigma-Aldrich). Then, the gel beads were washed with acetone (Sigma-Aldrich), and the solution was mixed vigorously and centrifuged. The gel beads were washed three times each with DPBS, followed by isopropanol. In the case of cell-DS, the encapsulated cells were further lysed using the lysis solutions in gel beads. First, 50 U/ µL Ready-Lyse Lysozyme Solution (Epicentre), 2 U/ mL Zymolyase (Zymo Research), 22 U/mL lysostaphin (MERCK), and 250 U/mL mutanolysin (MERCK) were added and incubated in DPBS at 37 °C overnight. Then gel beads were washed three times and 0.5 mg/mL achromopeptidase (FUJIFILM Wako Chemicals) was added into DPBS at 37 °C for 8 hours. The gel beads were washed with DPBS three times again and 1 mg/mL Proteinase K (Promega) with 0.5% SDS in PBS was added and incubated overnight at 40 °C. Following lysis, the gel beads were washed with DPBS five times. Then, the droplets in cell-DS and NP-DS were processed for multiple displacement amplification (MDA) using REPLI-g Single cell Kit (QIAGEN). The droplets were suspended in Buffer D2 from a REPLI-g Single Cell Kit and incubated for 2 hours or 8 hours for cell-DS or NP-DS, respectively. Following whole-genome amplification (WGA), gel beads were washed three times with 500 µL DPBS. Thereafter, beads were stained with 1× SYBR Green (Thermo Fisher Scientific) in DPBS. Following the confirmation of DNA amplification in the presence of green fluorescence in the gel, fluorescence-positive beads were sorted into 0.8 µL DPBS in 96-well plates using the FACSMelody cell sorter (BD Bioscience) equipped with a 488-nm excitation laser. Following droplet sorting, 96-well plates were proceeded to the second round of WGA. Second-round MDA was performed with the REPLI-g Single Cell Kit. The 96-well plates were incubated at 65 °C for 10 min after the addition of Buffer D2 (0.6 µL) to each well. Thereafter, 8.6 µL of MDA mixture was added to each well, and the plates were incubated at 30 °C for 2 hours for cell-DS and 8 hours for NP-DS, followed by the incubation at 65 °C for 3 minutes. Following second-round amplification, aliquots of the 96 well-plates were transferred to replica plates for DNA yield quantification using the Qubit dsDNA High Sensitivity Assay Kit (Thermo Fisher Scientific). For cell-DS, 16S rRNA gene Sanger sequencing (FASMAC) with the V3-V4 primers (Forward, 5′-TCGTCGGCAGCGTCAGATGTGTATAA GAGACAGCCTACGGGNGGCWGCAG-3′; reverse, 5′-GTCTCGTGGGCTCGGAGATGTGTATAAGAGAC AGGACTACHVGGGTATCTAATCC-3′) was also performed using the replica plates. The whole-genome amplified samples with enough DNA were further

subjected to the whole-genome sequencing analysis. In the case of cell-DS, the whole-genome amplified samples were further screened using the Geneious software (Biomatters, Ltd.) based on the quality of 16S rRNA with the criteria that more than 400 bp in length and HQ 40%. For whole-genome sequencing analysis, sequencing libraries were prepared from second-round MDA products using the Nextera XT DNA Library Prep Kit (Illumina) according to the manufacturer's protocols and sequenced using the HiSeq System (Illumina) 2 × 150 bp configuration for the biofilm samples or 2 × 75 bp configuration for *P. gingivalis* BEVs.

Estimation of the percentage of particles with DNA in cell-DS and NP-DS.

The theoretical ratio of droplets to particles in the encapsulation step is denoted as R_t and described as follows:

$$R_t = \frac{N}{t}$$

Here N is the total number of nanoparticles (cells) and t is the number of droplets. We used the fixed R_t for cell-DS or NP-DS as described above. Here, the nanoparticle samples subjected to the gel-lysis are considered as a mixture of viruses, BEVs, and other nanoparticles (extracellular proteins), and thus N can be described as follows:

$$N = \alpha + \beta + \gamma + \delta$$

Here, α, β, γ, and δ are the total numbers of viruses, BEVs with DNA, BEVs without DNA, and other nanoparticles. The observed ratio of droplets to particles with DNA in the encapsulation step is denoted as R_o and described as follows:

$$R_o = \frac{\alpha + \beta}{t}$$

For estimating R_o, the percentage of DNA-containing droplets (we call this parameter as P_o hereafter) after processing cells or nanoparticles were quantified by counting the number of droplets with green fluorescence in microscopic images. From P_o, we further estimated the ratio of droplets to particles with DNA (R_o) using the empirical curve in a previous study [2]. We can also estimate the ratio of BEVs (R_b) by positive particles with lipid-staining dye using NTA.

$$R_b = \frac{\beta + \gamma}{\alpha + \beta + \gamma + \delta}$$

The ratio of droplets containing viruses to those containing BEVs with DNA was estimated based on the taxonomic profiles (Fig. 4).

$$R_{vb} = \frac{\alpha}{\beta}$$

Then the percentage of BEVs with DNA (r) was calculated as follows.

$$r = \frac{\beta}{\beta + \gamma} = \frac{R_o}{R_t \cdot R_b(1 + R_{vb})}$$

Acquisition of transmission electron micrographs (TEM)

Purified BEV samples were placed onto formvar coated copper grids and negatively stained with 2% phosphotungstate acid solution (pH = 7.0) for 60 sec. The grids were analyzed and visualized using a JEM-1400 microscope (JEOL Ltd.) operated at an acceleration voltage of 100 kV and imaged using an EM-14830RUBY2 CCD camera (JEOL Ltd.).

Acquisition of scanning electron micrographs (SEM) of P. gingivalis cells

Pre-cultured *P. gingivalis* cells were first washed with PBS after centrifugation for 10 min at 7800 rpm and 25ºC. ITO glass (GEOMATEC) was immersed into the resuspended cell culture for 30 min with petroselinic acid (PA), artepillin-C (APC), or dimethyl sulfoxide (DMSO) at 25ºC. Thereafter, the ITO glass was removed from the culture, fixed with 2.5% glutaraldehyde, and washed three times in 0.1 M phosphate (pH 7.4) buffer. The washed ITO glass samples were subjected to a gradual dehydration process, wherein they were exposed to ethanol gradients of 25, 50, 75, 90, and 100% in the same buffer solution. Subsequently, the samples were exchanged thrice with t-butanol and freeze-dried under vacuum. The dried ITO glass was then subjected to a platinum evaporation coating process. and visualized using a JSM-7900F field-emission electron microscope (JEOL Ltd.) at an acceleration voltage of 2 kV.

Purification of BEVs from *P. gingivalis* after the petroselinic acid

P. gingivalis W83 was first grown in Gifu anaerobic medium at 37°C aerobically by pre-sparging of N2/CO2 (80:20 v/v) gas. After the incubation for ≈ 22 hours (OD600 reaches to approximately 0.5 ~ 1.0), the culture was centrifuged for 10 min at 7800 rpm and 25 °C and the resulting pellet was resuspended in the same amount of PBS as the original culture to remove pre-existing BEVs. Then the resuspended cell culture was treated with either of 64 μM petroselinic acid (PA) (FUJIFILM Wako Chemicals) or dimethyl sulfoxide (DMSO) (Sigma-Aldrich) incubated for 1 hour at 25ºC. Then, the culture was centrifuged for 10 min at 7800 rpm and 4°C The resulting supernatant was passed through 0.22 μm filters to remove any cell debris. The filtered supernatant was ultracentrifuged for 2 h at 126,000 × g at 4 °C and the resulting pellets were re-suspended in phosphate buffered saline (PBS).

Purification of BEVs from *P. gingivalis* cultured with artepillin-C

P. gingivalis W83 was grown in Gifu anaerobic medium at 37°C aerobically by pre-sparging of N2/CO2 (80:20 v/v) gas with either of 20 μM artepillin-C (APC) (FUJIFILM Wako Chemicals) or the same amount of DMSO (Sigma-Aldrich). After the incubation for ≈ 22 hours (OD600 reaches to approximately 0.5 ~ 1.0), the culture was centrifuged for 10 min at 7800 rpm and 25 °C and the resulting supernatant was passed through 0.22 μm filters to remove any cell debris. The filtered supernatant was ultracentrifuged for 2 h at 126,000 × g at 4 °C and the resulting pellets were re-suspended in phosphate buffered saline (PBS).

Quantification of DNA copy numbers by Real-time PCR analysis and nano flowanalyzer

For quantifying the amount of target DNA in the BEVs, (shown in Fig. 1B), the DNase I treated BEV samples from the *P. gingivalis* culture were subjected to the quantitative real-time PCR (qPCR) assays using Taqman Gene Expression Master Mix (Thermo Fischer Scientific). Briefly, qPCR assays were run in a dual color format utilizing TaqMan probes. The complement oligonucleotide probes to each target DNA fragments were prepared and labelled with FAM-TAMRA (see Supplementary Table 7, for the entire information of the primer and probe sequences used for this study). For the quantification of DNA contained in the BEVs, the standard curves were obtained by the serially diluted *P. gingivalis* genomic DNA samples (100 - 0.1 μg) extracted using Isoplant DNA extraction kit (NIPPON GENE). For sample preparation, BEV samples were pretreated with Proteinase K (Takara bio) for overnight at 37°C, then heated at 98°C for 20 min. 4 μL of treated samples were diluted to a 20 μL reaction mixture and subjected to following thermocycling steps: 10 min at 95°C, and 45 cycles of 15 s incubation at 95°C and 1 min incubation at 60°C using StepOnePlus™ Real-Time PCR System (Thermo Fischer Scientific). All samples were triplicated for all measurements. The results were analyzed with StepONE software v2.3 (Thermo Fischer Scientific).

The quantification of BEV concentrations subjected to the qPCR analysis were performed using nano flowanalyzer (NanoFCM). We stained the isolated nanoparticles with the lipid-staining dye, 33.3 μM DiD (1,1'-Dioctadecyl-3,3,3',3'-Tetramethylindodicarbocyanine) and DNA-staining dye, 2.67 μg/mL 1000×SYBR-Green I. The samples were diluted in PBS so that the total detected events were less than ≈ 10000 /min. The measurements were conducted with a sampling pressure of 1.0 kPa, and the side scatter, green fluorescence, and red fluorescence signals were detected by the irradiation of 488 nm and 638 nm lasers (set to 10 mW power and 10% decay for side scatter signals) with the bandpass filters (Green: 525/40; Red: 670/30 (peak wave length / half width)). A standard silica nanoparticle with a diameter of 250 nm (NanoFCM) was employed as a reference solution to determine the concentration of total nanoparticles. A standard silica nanosphere cocktail S16M-Exo (NanoFCM) was used for the size calibration. The NanoFCM Profession v2.0 software was used for data acquisition and processing. The total number of lipid-stained nanoparticles (BEVs) was determined using the ratio of the subpopulations estimated as red fluorescence signal positive and more than 50 nm in size by means of gating processes in the software.

Finally, the estimated copy number of target genomic regions in the qPCR was divided by the total number of BEVs, resulting in the average copy number of the target genomic region detected in a BEV particle in a given population.

Estimation of DNA fragments in extracellular DNA (eDNA) or BEV fraction from *P. gingivalis* culture using Real-time PCR analysis

Overnight-grown *P. gingivalis* cultures were prepared and centrifuged for 10 min at 7800 rpm and 4 °C. The resulting supernatant was passed through 0.22 μm filters to remove any cell debris. The filtered supernatant was ultracentrifuged for 2 h at 126,000 × g at 4 °C to separate extracellular and BEV fractions. The resulting pellets were re-suspended in PBS (we call this solution as the BEV fraction). The eDNA was collected from the extracellular fractions using ethanol precipitation. The 10 mL of the supernatant after

ultracentrifugation was collected and 3M sodium acetate at 1/10 volume and 100% ethanol of 2.5 volumes of the supernatant was added. The solution was incubated at –20ºC overnight. After the incubation, the solution was centrifuge at 4ºC at 15,000 rpm for 30 minutes and the supernatant was removed. Then, the resulting pellet was further washed with 100 µL of ice-cold 70% ethanol and centrifuged at 4ºC at 15,000 rpm for 5 minutes. The resulting supernatant was removed, and the pellet was air-dried at room temperature and then eluted with TE buffer. The collected DNA pellet was further purified with NucleoSpin Gel and PCR Clean-up kit (MACHEREY-NAGEL) and eluted with ultrapure water (we call this solution as eDNA fraction). As a control, we also prepared the DNA extracted from the BEV fraction. We first collected the suspended pellets equivalent to a 10 mL *P. gingivalis* culture and treated with DNase I and extracted DNA using the Isoplant-II DNA extraction kit (NIPPON GENE) according to the manufacturer's instructions. Then the amount of the targeted genomic regions was quantified using the quantitative real-time PCR analysis with the same configuration as described above. The estimated total amount of the targeted DNA fragments was normalized to be the quantity obtained from 1 mL of the *P. gingivalis* culture.

Identification of terminal inverted repeats (TIRs)

Identification of TIRs from the target genome region was performed using RepeatScout and RepeatMasker [4]. First, the "RepeatScout" and "build_lmer_table" algorithms were used to identify frequently occurring k-mers within the target genome region. The -lmer option is set to 12 and 14 for transposons and the other longer DNA regions, respectively. The sequences shorter than each set -lmer option were excluded from the analysis because shorter TIRs have not been reported in each case [5, 6]. Then the repeat sequences were comprehensively searched in the target genomic region by the "RepeatMasker". For transposon case, the 500 bp upstream or downstream regions from the ORF site was targeted, and pairs of repeats that flank the target ORF and are complementary each other were further screened by the custom code. For other genomic loci, the pairs of repeats that flank more than 5 kb region were further screened. If the identified TIRs exist in close proximity to each other, such TIRs were merged into a single TIR manually.

Computation of cumulative GC skewness and identification of *dif* sites

Using the number of nucleotide G and C (nG and nC), GC skewness was calculated using as follows:

$$GCskew = \frac{nG - nC}{nG + nC}$$

For a 10 kb genomic region in a target genome, GC skew was calculated in 200 bp windows, and the average value was used as the GC skewness for each 10 kb region. The cumulative sum from the initial position of the genome sequence was calculated and plotted as cumulative GC skewness in Supplementary Fig. 9. The sequence information and genomic positions for *dif* sites were referred to the previous study [7].

Pangenome analyses of *Porphyromonas*

The bacterial genomes grouped in the *Porphyromonas* genus were first collected from GTDB [8] (version r214) and subject to the completeness and contamination checks using CheckM 1.1.3 [9], resulting in 244 genomes that showed > 90% completeness and < 5% contamination from 41 species (Supplementary Data 4). The CDSs in those genomes were extracted using Prokka 1.14.6 [10], and the extracted CDSs were grouped into orthologous groups using Orthofinder 2.4.0 [11] with the option "-og". We constructed a maximum likelihood phylogenetic tree in the *Porphyromonas* group using the bacterial marker genes collected using GTDB-tk (version 2.1.1) [8]. For the 244 genomes in *Porphyromonas*, a multiple sequencing alignment (MSA) of the marker genes was first performed using the "align" command in GTDB-tk. A phylogenetic tree reconstruction using iqtree 2.0.3 [12] was performed on the resulting MSA file from GTDB-TK. The best-fitting model of sequence evolution for the given MSA was searched using ModelFinder [13]. The estimated phylogenetic and gene-cluster tree visualizations were performed using the ETE toolkit [14].

Construction of gene-cluster trees

For a target gene cluster, we first extracted orthologous genes (CDS) for each gene from 244 genomes based on the OrthoFinder results. Then, each orthologue gene set was aligned using MAFFT 7.5.20 [15]. The poorly conserved regions were removed from the resulting MSA files using trimAl 1.4.1 [16] with options "-automated1". Then, the alignments of all orthologues in the target gene cluster were concatenated and used for tree construction using iqtree with ModelFinder as mentioned above.

Screening phylogenetically discordant genes

Orthologous gene sets whose pairwise similarities were significantly discordant with the average pairwise similarities of the genomes are candidates of horizontally transferred genes, and such phylogenetically discordant gene sets were screened using BLASTP scores based on Clarke *et al.* [17]. We targeted 1579 orthologous gene sets identified using OrthoFinder, which consist of more than 25 genes (10% of all analyzed genomes). In each orthologue group, protein homology searches against the protein sequences from all-gene members were performed using DIAMOND using the gene from *P. gingivalis* W83 (GCF_000007585.1) as a query sequence. The genes belonging to multiple orthologous groups were excluded from the analysis. The bit-scores of those homology searches were normalized by the self-matching score (i.e., the bit-score against the protein sequence from *P. gingivalis* W83), and these normalized bit-score (u) were used as metrics of similarities in each orthologous group. If multiple gene members from an identical genome exist in the same orthologue group, the gene with the highest bit-score was used for the analysis. Finally, in an orthologue group O, we obtained the array of the normalized bit-scores as follows:

$$U_O = \{u_{O,1}, u_{O,2}, \cdots, u_{O,G}\}$$

Here, $u_{O,G}$ is the normalized bit-score of a gene from the genome *G* that belongs to the orthologue group *O*. Then, for each target genome *G*, we can also obtain an array of the normalized bit-scores as follows:

$$U_G = \{u_{1,G}, u_{2,G}, \cdots, u_{O,G}\}$$

The median of the U_G (denoted as w_G) is used as a metric of the overall sequence similarity for a target genome *G* to the query genome (GCF_000007585.1), then we obtained the array of the overall sequence similarity for the targeted genomes as follows:

$$W = \{w_1, w_2, \cdots, w_G\}$$

By checking Pearson's correlation between U_O and *W* in each orthologue group, we determined whether the sequence similarities in that gene set are discordant with the overall similarities of the target genomes. To test whether the observed correlation is significantly low (i.e., the correlation is significantly lower than the expectation), we performed the permutation test. For the genomes whose genes are involved in the target orthologue group, we randomly picked out *u* from U_G array and generated a random set of normalized bit-score and computed Pearson's correlation to *W*. We repeated this process 10,000 times, and the probability that the correlation was higher than the observed value was computed. If this probability is lower than 0.05, that orthologue group is regarded as phylogenetically discordant gene sets with overall genome sequences, and thus possibly horizontally transferred genes.

Finding possible HGT events of the target gene cluster in *Porphyromonas*

The detection of possible HGT events in the target gene cluster was performed by reconciling phylogenetic (species) and gene-cluster trees using RANGER-DTL 2.0 [18]. The phylogenetic and gene-cluster trees were first collapsed at the species level using ETE tool kit. Then, RANGER-DTL module was run with the following cost parameter set (-D 2 -T 5 -L 1) to reconcile the trees for 10,000 times. Transfer events detected in more than 50% of the reconciliation analysis were labeled as HGT events between species.

Pre-processing of sequence reads and extraction of CDSs in droplet sequencing

The paired-end read sequences from each droplet were first processed using fastp 0.19.5 [19] for quality control and adapter trimming. The processed sequence reads were assembled using SPAdes 3.12.0 [20] with options (-k auto –disable-rr --careful). Then CDSs, rRNAs, and tRNAs were extracted from the contigs by Prokka 1.14.6 [10].

Taxonomic annotation of CDSs in droplet sequencing

The extracted CDSs from droplets derived from the same sample were grouped together and clustered using CD-HIT [21] with options (-c 0.98 -s 0.5 -aS 0.9). The clustered CDSs were first annotated by a

homology search against the National Center for Biotechnology Information (NCBI) non-redundant (nr) database (downloaded on July 1st, 2021) using DIAMOND 2.0.8 [22] with options (--evalue 1e-10 –outfmt 6 –sensitive). For each CDS, the Genbank accession number of the best-hit protein sequence was further used for taxonomic annotation using BASTA [23] with options (sequence prot -l 100 -I 80 -b). At this step, hits whose percent identity is less than 80% and whose matched length is less than 100 bp were discarded. Genbank accession numbers that were not mapped to the default BASTA database were further mapped to the NCBI database using custom python code implemented in the original BASTA pipeline. Briefly, taxonomic information of each protein sequence in DIAMOND hits was obtained using Bio.Entrez module in Biopython package [24] and formatted using TaxonKit [25].

The CDSs classified as Bacteria in the kingdom-level were further annotated using a homology search against protein sequences in the Genome Taxonomy Database (GTDB) [8] (version r202) using DIAMOND for more strict taxonomic annotation of bacterial CDSs. If a top-hit of CDS to the GTDB protein database with a percent identity ≥ 80%, the assembly accession number registered in GTDB was assigned. We eliminated bacterial taxa that were classified as potential contaminants in low-biomass human samples in previous studies [26, 27]. The list of eliminated GTDB taxa is shown in Data. S2.

Screening of most frequently detected taxon in BEV-containing droplets

For droplets determined to BEV-containing, we first grouped CDSs using their taxonomic information (GTDB accession number) and calculated the total CDS length assigned to each GTDB taxonomy. Then the most abundantly detected GTDB taxonomy in the total CDS length is designated to the "most frequently detected taxon" (MFT) for each droplet.

Identification of detected and enriched genomic regions in BEVs

We first aligned sequence reads obtained from each droplet to the assembly genomes of host bacteria using bowtie2 [28] with option (--sensitive). We analyzed BEV-containing droplets from the *P. gingivalis* and mapped the sequence reads to the host bacterial genome (*P. gingivalis*: GCF_000007585.1). In the case of dental biofilm samples, we focused on *Alcaligenes faecalis* (GCF_002443155.1), which were frequently detected as MFT in droplets. It is well known that MDA usually harbors chimeric sequences during the amplification process [29] and it is possible that presence of those chimeric reads affect the mapping profiles. We checked this possibility using BWA [30] and found that only 1.41 ± 0.34% of reads were classified as chimeric in 96 droplets (Supplementary Fig. 20). We also confirmed that cleaning of those chimeric reads using recently developed algorithm [31] did not harbor qualitative difference in the mapping profiles compared to those generated from the original read sequences (Supplementary Fig. 20).

Then, the assembly genome sequences for each bacterium were separated into 1000 bp sections.
Each section is regarded as detected (i.e., filled with black in Fig. 2B), when almost the entire area (> 99%) was mapped by reads more than 1 time. For identifying the contiguous regions that mapped by reads, 2 regions where the start position of one region and the end position of the other region are less than 2 kbp

distant were regarded to be adjacent and treated as a single contiguous region, otherwise the 2 regions were derived from different loci (i.e., the separate fragments). In the case of *P. gingivalis,* identical genomic regions, especially those in IS elements, appeared multiple times, which potentially leads to the mapping of the sequence reads to broader regions than their actual extent. To exclude this effect for calculating the length of all detected regions and continuous regions, we excluded the IS elements (Supplementary Data 2) for the calculation of the total detected region or continuous genomic region in each droplet.

Significantly enriched genomic regions in the droplets were screened using a binomial test with the following formula:

$$p = \sum_{i=k}^{n} \binom{n}{i} {p_0}^{k} (1 - p_0)^{n-k}$$

Where n is the total number of droplets examined, k is the number of positive droplets (i.e. droplets in which the targeted 1000 bp section was detected), and p_0 is the average frequency of positive droplets for the overall genomic region. If p (P value) is less than 0.01, that 1000 bp section is regarded as an enriched genomic region in BEV-containing droplets.

Gene enrichment analysis in BEV-containing droplets

The significantly enriched genomic regions were divided into CDS-unit using Prokka and functionally annotated using a homology search using DIAMOND against UniprotKB/Swiss-Prot database [32] (Downloaded on April 21, 2021). To see the enrichment of specific functional categories in BEV-derived CDSs, the gene ontology (GO) annotations [33] are attributed to a top hit in UniprotKB/Swiss-Prot database assigned to each CDS. Then enriched GO annotations in BEV-derived CDSs were screened using a hypergeometric test with the following formula:

$$p = \sum_{i=k}^{n} \frac{\binom{G}{i}\binom{G-g}{n-i}}{\binom{G}{n}}$$

Where n is the total number of enriched CDSs, G is the total number of CDSs in the genome, and g is the total number of CDSs in a GO annotation category of interest. The probability that more than k CDSs were found in each GO annotation category is denoted as p. If p is less than 0.05, that GO annotation is regarded as an enriched functional category in BEV-containing droplets.

Supporting Figures

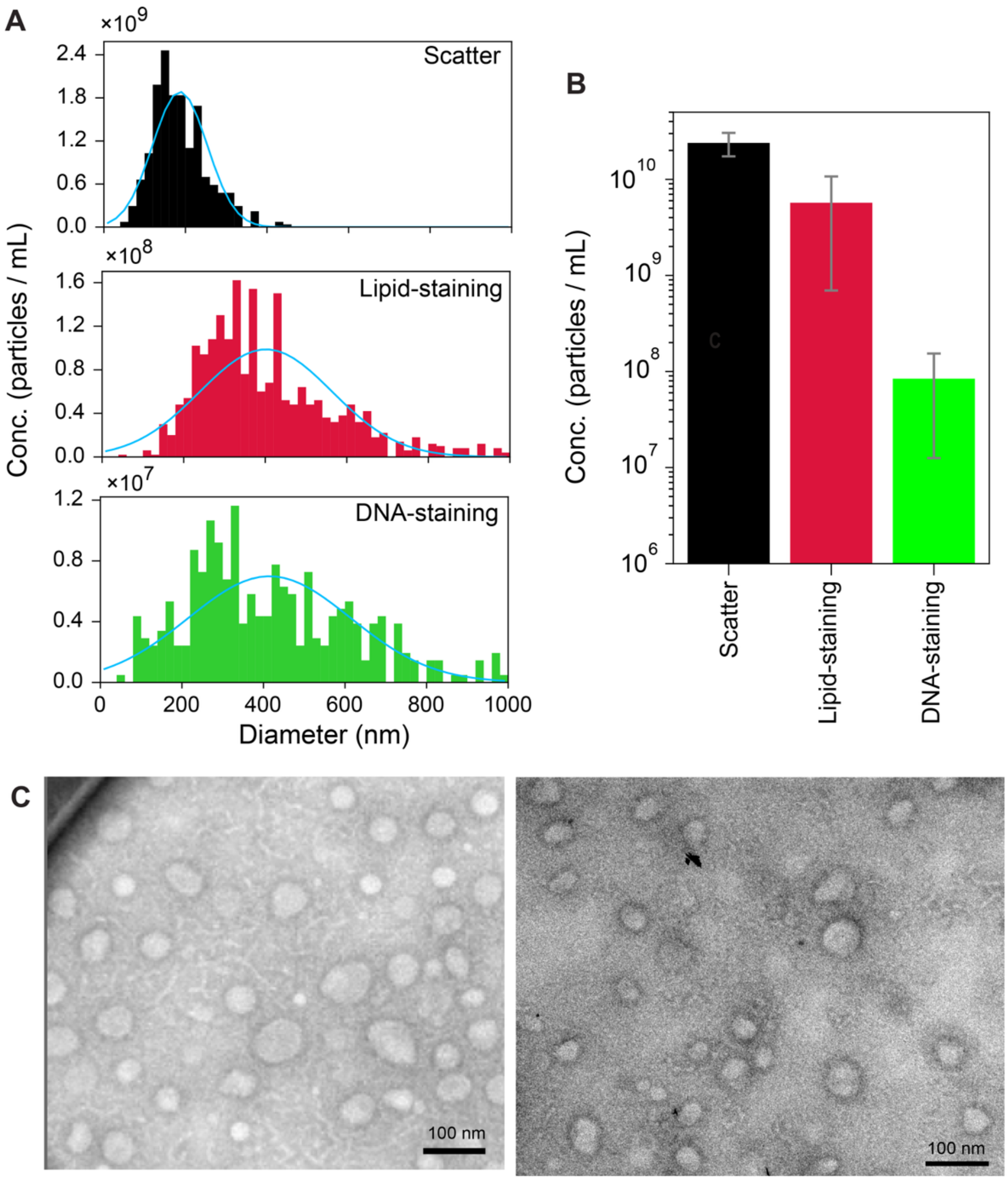

Supplementary Figure 1. NTA (nanoparticle tracking analysis) of BEVs isolated from pure cultures of *P. gingivalis*. (A) The typical size distribution of nanoparticles isolated from pure cultures of *P.gingivalis* using NTA (nanoparticle tracking analysis). We stained the isolated nanoparticles with the lipid-staining dye, 33.3 μM DiD (1,1'-Dioctadecyl-3,3,3',3'-Tetramethylindodicarbocyanine), and DNA-staining dye, 2.67 μg/mL 1000×SYBR-Green I. We detected the scattered light signals (Scatter, black), lipid dye fluorescence (red, detected in 660/680 nm laser-filter unit), or DNA dye fluorescence (green, detected in 488/500 nm laser-filter unit). A blue line is the fitted curve of the normal distribution to the experimental data. (B) The total concentration of nanoparticles detected by each signal with 50 ~ 500 nm in size. Error bars indicate the standard deviations in triplicate experiments. (C) The typical TEM micrographs of the purified nanoparticles from the *P. gingivalis* culture.

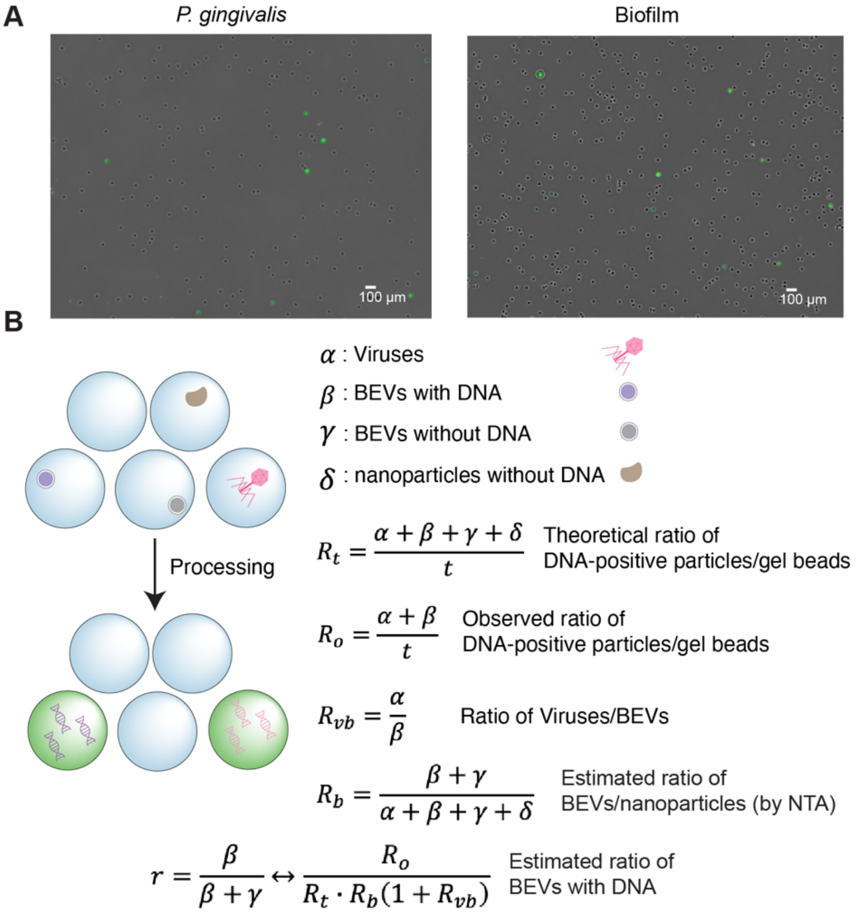

C

	R_o	R_t	R_{vb}	R_b	r
P. gingivalis	0.0462	0.361	0	0.206	0.622
Biofilm	0.0352	0.361	3.97	0.0553	0.335

Supplementary Figure 2. The ratio of droplets containing amplified DNA fragments after processing cells or nanoparticles. (A) Representative microscopic fluorescence images of gel beads after in-gel lysis and amplification. The beads were stained with SYBR-Green-I to check the number of positive droplets (i.e., internal DNA was successfully amplified). (B) Schematic of estimating the percentage of nanoparticles with DNA. The nanoparticles expected to contain DNA, BEVs with DNA or phages, were colored by purple and red, respectively. Gel beads expected to contain amplified DNA fragments were colored green. The theoretical ratio of droplets to total particles (R_t) was controlled to a fixed value in our analysis (see Supporting Materials and Methods). The ratio of droplets to particles with DNA (R_o) was estimated by the percentage of positive droplets in a microscopic image and an empirical curve in a previous study. Based on those two parameters and other two parameters estimated using NTA and taxonomic annotation (see Supporting Materials and Methods), we calculated the percentage of BEVs with DNA, *r*. (C) The observed percentage of positive droplets and estimated percentage of BEVs with DNA.

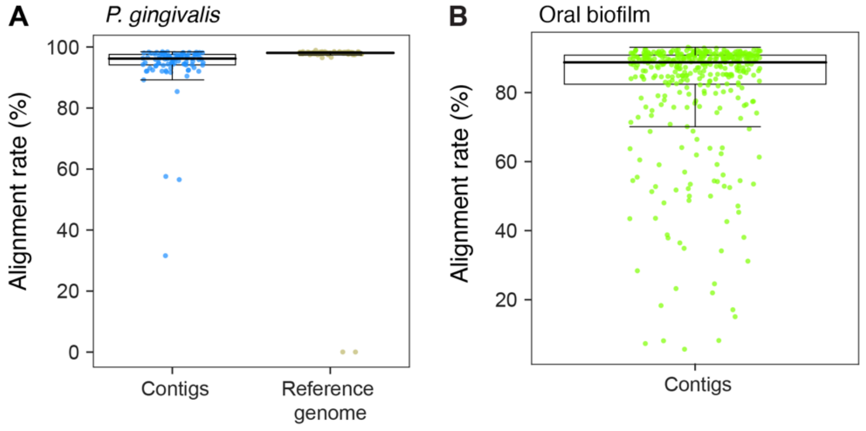

Supplementary Figure 3. Alignment rate of the sequence reads to the assembled contigs or reference genome in NP-DS. (A) The results in the NP-DS of *Porphyromonas gingivalis* BEVs. For each set of sequence reads from each droplet, we performed the mapping using bowtie2 to the assembled contigs or the original bacterial genome (GCF_000007585.1). Then the percentage of the aligned sequence reads were plotted for each NP-DS. (B) The result in the NP-DS of the oral biofilm samples. The processed sequence reads were mapped to the assembled contigs for each droplet. Each dot corresponds to a result of a single droplet. In both cases, we mapped the sequence reads processed by fastp. The bold lines indicate the median values.

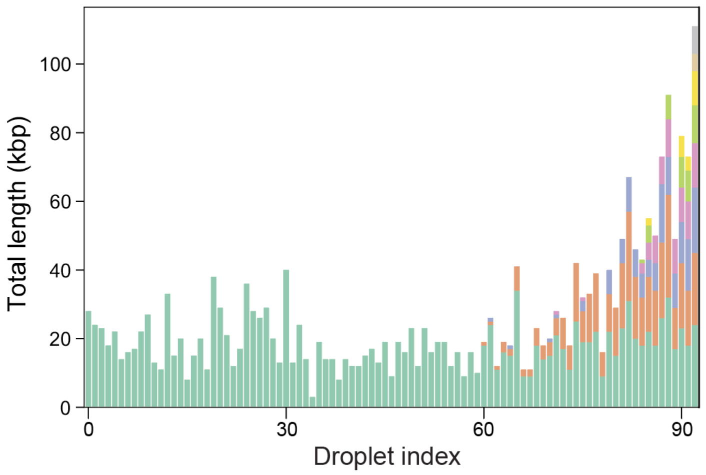

Supplementary Figure 4. DNA length of all detected fragments in each droplet containing *P. gingivalis* BEV. The lengths of detected contiguous genomic regions are plotted for each droplet. Stacked bar plots of different colors indicate DNA fragments originating from distant regions (> 2 kb) of the genome. Droplets are sorted in ascending order according to the ratio of the longest fragment length to the total detected region. Here, the results from 93 droplets, where more than 90% of sequence reads were mapped to the *P. gingivalis* genome, were shown.

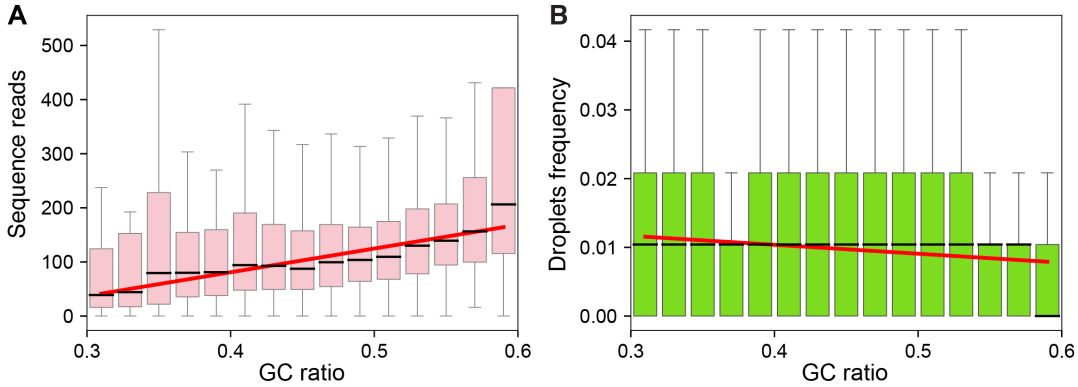

Supplementary Figure 5. GC content of genomic locus biases the frequency of sequence reads in bulk-BEV sequencing. (A) The difference in mapped sequence reads is dependent on the GC content of genomic loci in bulk-BEV sequencing. The genome of *P. gingivalis* W83 (GCF_000007585.1) was separated in 400 bp sections, and the average number of mapped sequence reads in each section was estimated. Those sections were binned by GC content (from the range of 30% to 60% separated by 2% intervals), and the mapped sequence reads were plotted as boxplots. (B) The relationship between the GC content and frequency of positive droplets on the genome of *P. gingivalis* in the NP-DS. Same as panel A, the *P. gingivalis* genome was separated by 400 bp sections and binned by GC content. The frequency of positive droplets in the binned groups are plotted as box plots. In both panels, red lines indicate the linear regression of the median values.

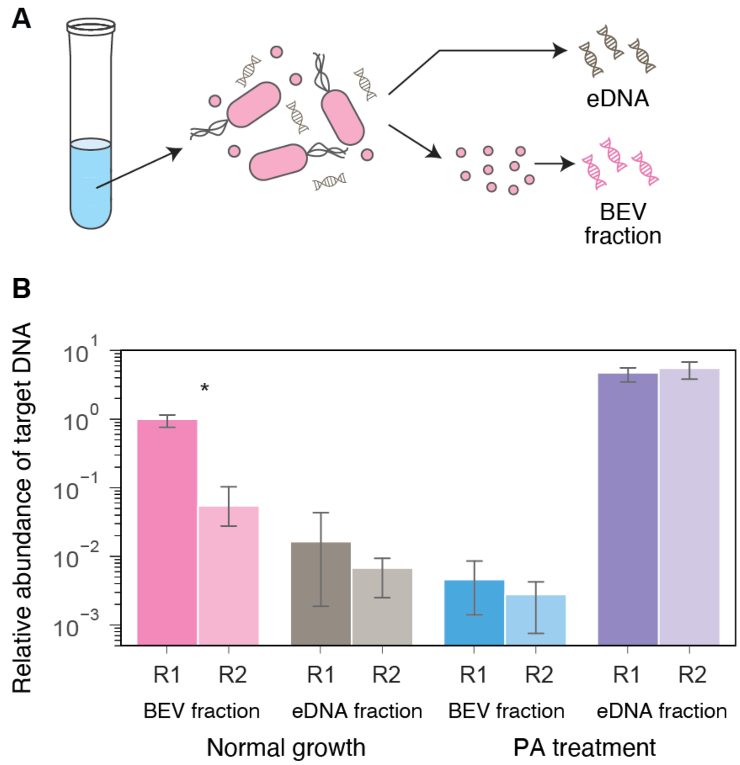

Supplementary Figure 6. The distinct characteristics of the extracellular DNA compared to the DNA from the BEV fractions in the *P. gingivalis* culture. The relative abundance of DNA fragments of the two target genomic regions in the eDNA or BEV fractions quantified using a real-time qPCR same as Fig. 1B. The DNA was purified from the extracellular space (eDNA) or BEV fraction in the equivalent volumes of the cultures. Here, we normalized the detected amount of DNA fragments by dividing the average value of the BEV fraction in the Region 1 (i.e., the enriched region in the BEVs) in the normal growth case (without PA treatment). The error bars indicate the standard deviations. Asterisks indicate statistical significance levels according to Student's t-test (*: P value < 0.05 and no symbol in not significant case).

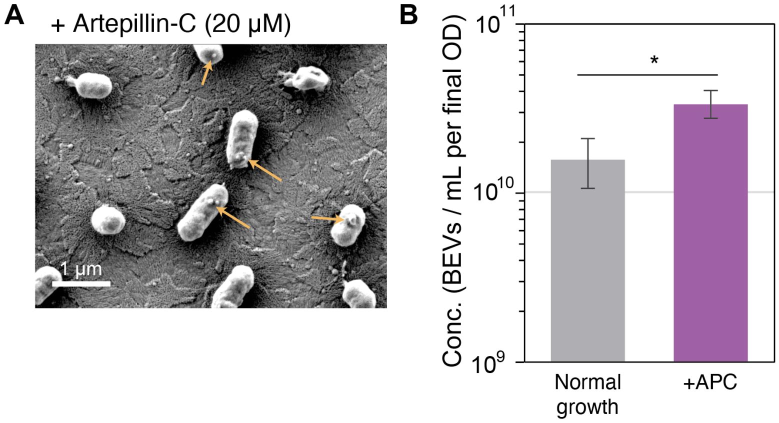

Supplementary Figure 7. The blebbing-promoting drug increased the BEV production level. (A) Scanning electron microscope (SEM) images of *P. gingivalis* cells treated with 20 μM Artepillin-C (APC) same as Fig.1 1E. (B) Total number of the lipid-dye-stained nano particles after 22 h of incubation with APC or DMSO, which was quantified using a nano flowanalyzer. Asterisks indicate statistical significance levels according to Student's t-test (*: P value < 0.05).

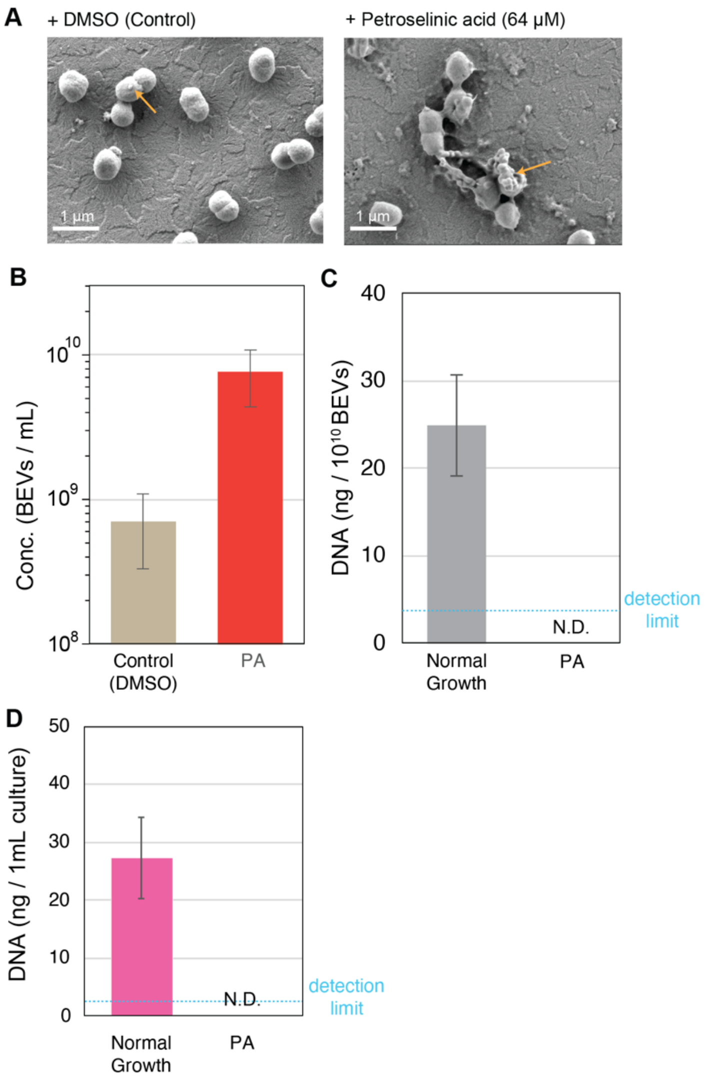

Supplementary Figure 8. Explosive cell lysis promotes BEV production but decreases the DNA content of BEVs in *P. gingivalis.* (A) SEM images of *P. gingivalis* cells treated with 64 μM Petroselinic acid (PA) or dimethyl sulfoxide (DMSO). The image of PA is same as Fig. 1E. (B) Total number of lipid-dye-stained nano particles after treatment with PA and DMSO, and quantified using a nano flowanalyzer. (C) The total DNA content purified from the BEV population after treatment with PA or that from naturally occurring BEVs (i.e., produced during the growth condition without drug treatment). The collected BEV samples were treated with DNase I and subsequently subjected to DNA extraction. The amount of DNA was normalized to the total number of BEVs. (D) Total amount of extracted DNA from BEVs from 1mL volume of cultures in the normal growth condition or the presence of PA. Error bars indicate standard deviation in triplicates.

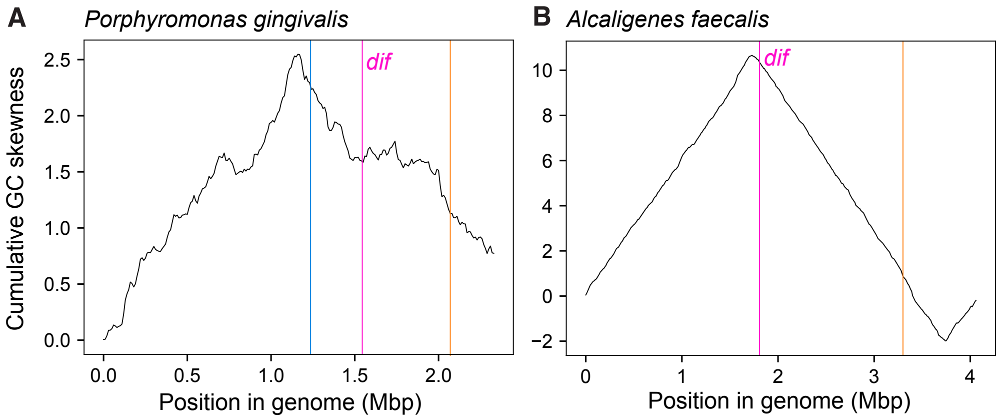

Supplementary Figure 9. The GC skewness and the *dif* site position on the targeted bacterial genomes. The cumulative GC skew scores were plotted as black lines. The magenta lines indicate the position of the *dif* site on each genome. (A) *P. gingivalis* W83 (GCF_000007585.1). Blue and orange lines correspond to the enriched gene clusters, cobalamin biosynthesis and Type III-B CRISPR-Cas, respectively. (B) *Alcaligenes faecalis* (GCF_002443155.1). The orange line corresponds to the enriched genomic position, O-antigen biosynthesis gene cluster.

```
>cobalamin_cluster_TIR1
1230980 CATAAGGCACCCGC
1248954 CATAAGGCACCCGC
        **************

>cobalamin_cluster_TIR2
1231083 TCAAGCTATATATAAATGGAAAACGATCTGTATATTTATTGCAAATGATTTATATATAAA
1248853 CCGATCTATATATAAATGGAAAACGATTTATATATAAAACGAAAACGATCTATATATAGA
         * * ********************** * *****  *  * *** *** ******** *

1231143 TCGTTTC
1248793 TCGAAAA
        ***

>cobalamin_cluster_TIR3
1231200 TCTTCGACTTCAAAATCCGCTTCCATTCAGGTGCTTATTTGGAATCATTCCAAACTCGTC
1248713 TTTCAGACTTCAAAATCCGCTTCTTTTTACCCCTTATTTAGAATCATTCCAAACTCGTC
        * *  ******************  ** *    ******* *******************

1231260 TTCGTTGTTGAAAACTTTTTTGGCTCGT
1248652 TTCGTTGTTGAAAACTTTTTTCGCGCGA
        ********************* ** **

>cobalamin_cluster_TIR4
1236244 GAACGATACGCTCA
1248045 GAACGATACGCTCA
        **************

>cobalamin_cluster_TIR5
```

```
1236763 TCCGTTTTCGGCAAT
1263507 TCCGTTTTCGGCAAT
        ***************
```

```
>cobalamin_cluster_TIR6
1237429 CGGTAAGACTGTTGCATGGGAGTTTCATTGAGCTCTTTTGCTGCAGAGCTGATTCTTAGT
1265343 AAATGAGACTGTTGCATGGGAGTTTGATTGAGCTCTTTTGCTGCAGAGCTGATTCTTAGT
           * ******************** **********************************

1237489 GTCTTCGGGAAAGGTCAAACCTCCGGTATATGGGTACCGAGCAAATAGAAATTTCCCCAA
1265283 GTCTTCGGGAAAGGTCAAACCTCCGGTATATGGGCACCGAGCAAATAGAAATTTCCCCAA
        ********************************** *************************

1237549 GTTTCCATCAGAGAAGTACTCCTTTCCTCGTCAAATAGGCGAGAAATAAGAAACGATTGT
1265223 GTTTCCATTAGAGAAGTACTCCTTTCCTCGTCAAATAGGCGAGAAATAAGAAACGATTGT
        ******** ***************************************************

1237609 CAGCTGTTTCTTGCTCCCTGCACGATGCAGGACGCGATTGTCAGTTGATTCTTGCTCCCT
1265163 CAGCCGTTTCTTGCTCCCTGCATGATGCAGGACGCGATTGTCGGCTGTTTCTTGCTCCCT
        **** ***************** ******************* * ** ************

1237669 GCACGATGCAGGACGCGATTGTCAGTTGATTCTTGCTTCCTGCACGATGCAGGACGCGAT
1265103 GCATGATGCAGGACGCGATTGTCGGCTGTTTCTTGCTCCCTGCATGATGCAGGACGCGAT
        *** ******************* * ** ******** ****** ***************

1237729 TGTCAGTTGATTCTTGCTCCCTGCACGATGCAGGACGCGATTGTCAGTTGATTCTTGCTC
1265043 TGTCGGCTGTTTCTTGCTTCCTGCACGATGCAGGACGCGATTGTCAGCTGATTCTTGCTT
        **** * ** ******** **************************** ***********

1237789 CCTGCACGATGCAGGACGCGATTGTCAGTTGATTCTTGCTTCCTGCACGATGCAGGACGC
1264983 CCTGCACGATGCAGGACGCGATTGTCAGCTGATTCTTGCTTCCTGCACGATGCAGGACGC
        **************************** *******************************

1237849 GATTGTCAGTTGATTCTTGCTTCCTGCACGATGCAGGACGCGATTGTCAGTTGATTCTTG
1264923 GATTGTCAGTTGATTCTTGCTTCCTGCACGATGCAGGACGCGATTGTCGGCTGATTCTTG
        ************************************************ * *********

1237909 CTTCCTGCACGATGCAGGACGCGATTGTCAGCTGACTCTGCTCCCATCAATACGCTAACT
1264863 CTTCCTGCACGATGCAGGACGCGATTGTCAGCTGATTCTGCTCCCATCAATGCGCTAACT
        *********************************** *************** ********

1237969 ATCAGCTGTTTGCAACTATTTTATAGGACTTTCATTGAAGTCTTTTGCCGCAGAGCTGAT
1264803 ATCAGCTATTTGCAACTATTTTATAGGACTTTTATTGAAGTCTTTTGCCGCAGAGCTGAT
        ******* ************************ ***************************

1238029 TCTTAGTTTTTTTTCAGATCAGGCAA
1264743 TCTTAAGTGTTTTTCAGATTACTTGA
        *****  * ********** *    *
```

```
> typeIII_CRISPR_cluster_TIR1
2040185 GAGATATTCGGACAG
2053190 GAGATATTCGGACAG
        ***************
```

```
> typeIII_CRISPR_cluster_TIR2
2041832 ATATGGCTTCGTCC
2057704 ATATGGCTTCGTCC
        **************

> typeIII_CRISPR_cluster_TIR3
2053628 GCAGTCGCTCAAGT
2068919 GCAGTCGCTCAAGT
        **************

> typeIII_CRISPR_cluster_TIR4
2060409 GCCCTCGACGAGGA
2079505 GCCCTCGACGAAGA
        *********** **

> typeIII_CRISPR_cluster_TIR5
2063556 GCCCTCGACGAGGA
2079505 GCCCTCGACGAAGA
        *********** **


> O-antigen_biosynthesis_TIR
3282151 TCCACCTTCATCGT
3309992 TCCACCTTCATCGT
        **************
```

Supplementary Figure 10. The identified possible TIRs adjacent to the two clusters in *Porphyromonas gingivalis* and *Alcaligenes faecalis.* The displayed DNA sequences here correspond to those shown in Fig. 2. Asterisks indicate the aligned nucleotide pairs between left-arm and right-arm of the inverted repeats. We screened TIRs whose length is more than or equal to 14 bps and the divergence score in RepeatMasker is less than 10.

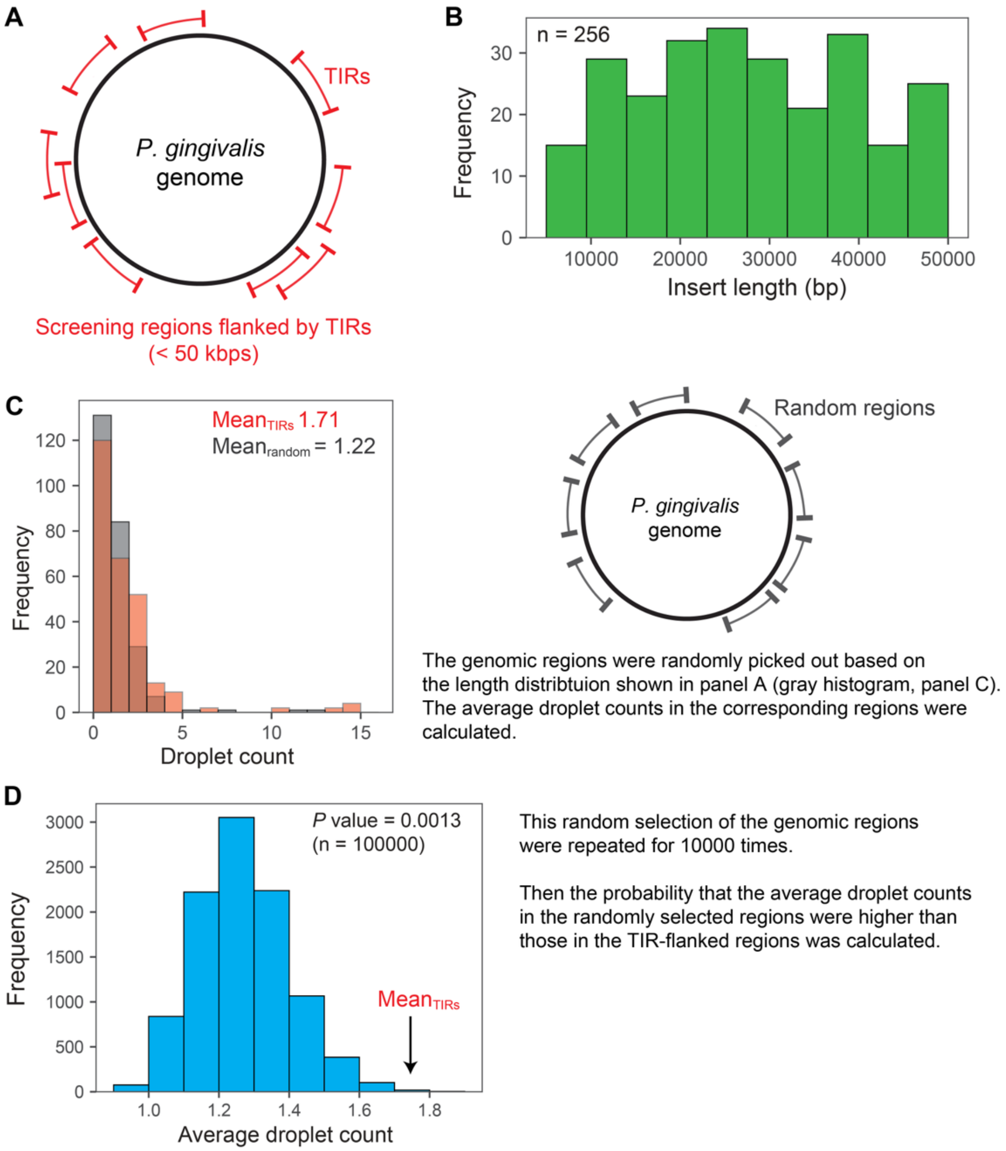

Supplementary Figure 11. Significant overrepresentation of the TIR-flanked genomic regions on the *Porphyromonas gingivalis* BEVs. (A) TIRs whose flanking regions are less than 50 kbp and more than 5 kbp were comprehensively screened. (B) The histogram of the insert length flanked by identified TIRs. We screened 256 TIRs on the *Porphyromonas gingivalis* genome. (C) The histogram of the average droplet counts in the TIR-flanked regions (red). We also randomly picked out the 256 genomic loci based on the empirical length distribution (panel B). A typical histogram of the average droplet counts for the randomly selected regions was shown (gray). Mean value of the droplet counts among the targeted regions were also shown. In the case of the randomly selected region, a mean value for one typical case is shown. (D) The histogram of the average droplet counts for the randomly selected 256 genomic loci whose length are subject to the distribution in panel B. The random selections were iterated for 10000 times, and the probability that the average droplet counts in the randomly selected regions were higher than those in the TIR-flanked regions (Mean$_{TIRs}$) were calculated and shown as *P* value.

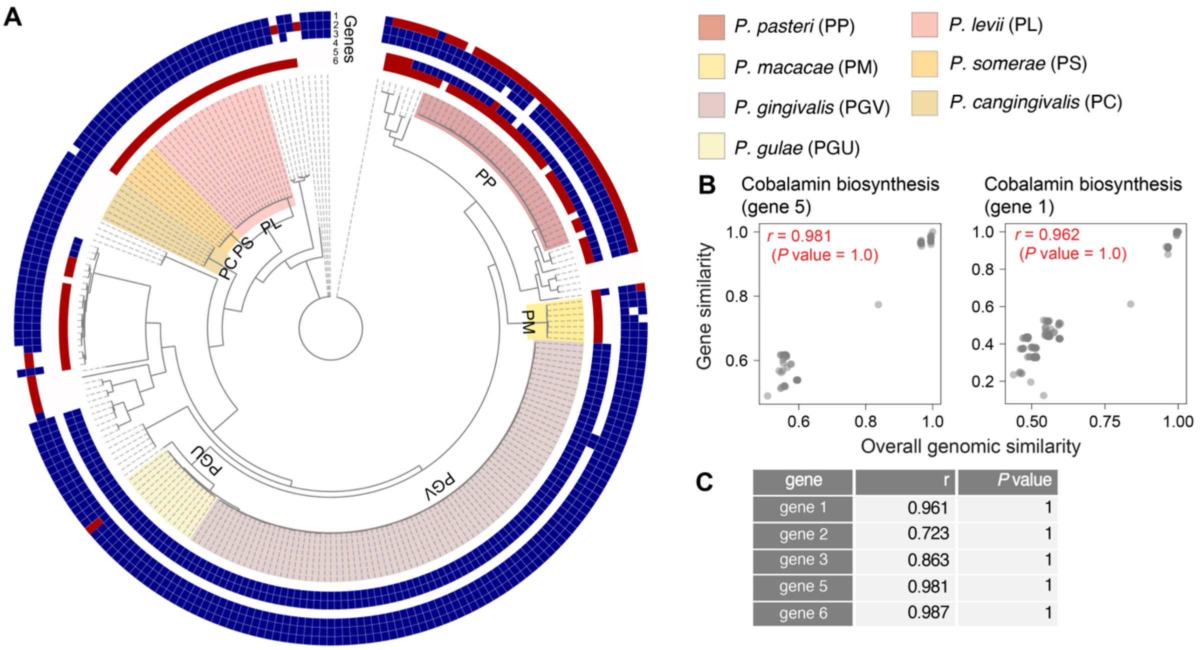

gene	r	P value
gene 1	0.961	1
gene 2	0.723	1
gene 3	0.863	1
gene 5	0.981	1
gene 6	0.987	1

Supplementary Figure 12. Prevalence and phylogenetic relationship of the cobalamin synthesis gene-cluster among *Porphyromonas* group. (A) Prevalence of the seven genes in the cobalamin biosynthesis gene cluster among 244 genomes in the *Porphyromonas group*. The presence of seven genes (displayed in Fig. 2) was shown as a heatmap. The genes that exist and are clustered on the genome are filled with blue, and those located out of the cluster are filled with red. The major species groups are shaded by colors in the phylogenetic tree. (B) Strong correlation between the overall similarity of the genome and the similarity of the genes in the cobalamin biosynthesis gene cluster. Pearson's correlation (*r*) between two parameters is displayed in each panel with the *P* value: the probability that the randomly picked out gene similarity score exhibited a lower correlation than the observed value in the permutation test (see Supporting Materials and Methods). (C) The correlation between the overall similarity of the genome and the similarity of the genes in all analyzed members. The numbers correspond to those in Fig. 2. The statistical significance levels of the correlation values are displayed as *P* values. The gene 4 were not analyzed here as they did not meet the criteria of the analysis.

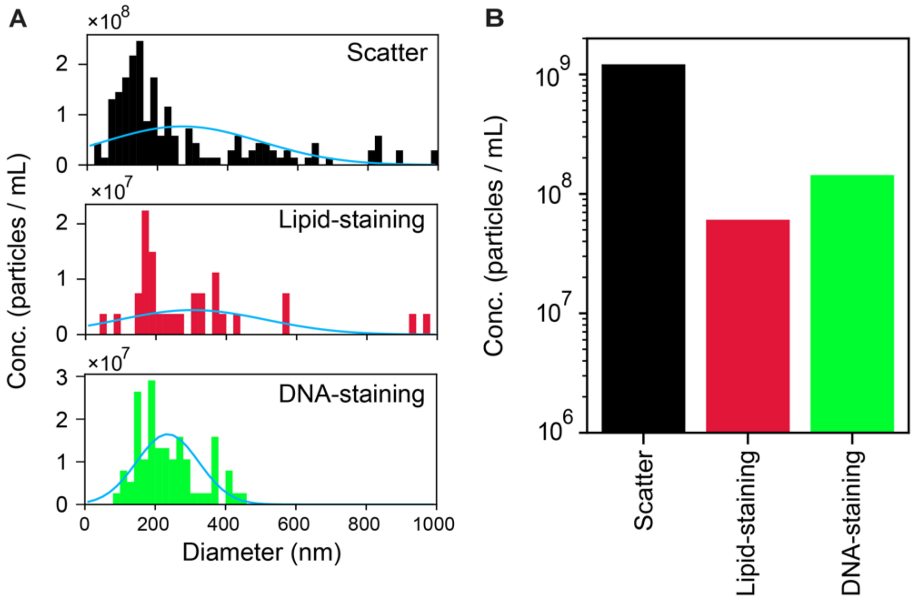

Supplementary Figure 13. Characterization of isolated nanoparticles from the dental plaque biofilm. (A) The size distribution of nanoparticles isolated from the dental plaque sample. The samples were stained with 5 μg/mL lipid staining FM4-64 for and 1 μg/mL of 1000×SYBR Green I. We detected the scattered light signal (black), lipid dye fluorescence (red, detected in 660/680 nm laser-filter unit), or DNA dye fluorescence (green, detected in 488/500 nm laser-filter unit) using NTA. A blue line is the fitted curve of the normal distribution to the experimental data. (B) The total concentration of particles detected by each signal with 50 ~ 500 nm in size.

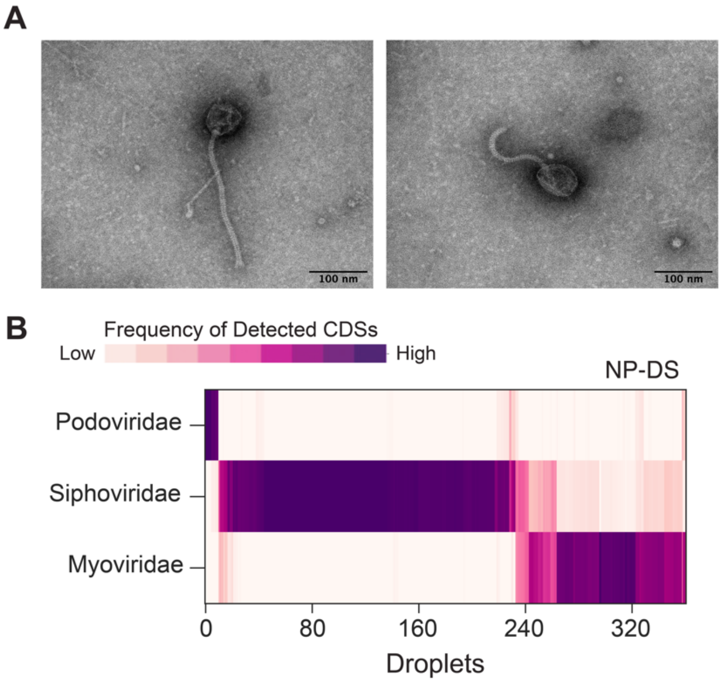

Supplementary Figure 14. Phage particles in an isolated nanoparticle sample from the dental plaque. (A) The typical TEM micrographs of phage-like particles isolated from the dental plaque of periodontal patients. (B) Taxonomic profiles of phage-derived CDSs detected in NP-DS. Class-level taxonomic classification of viral CDSs (vCDSs) detected in NP-DS was shown as a heatmap. The color of the heatmaps showed the length of CDSs assigned to each taxonomy relative to the total length of vCDS detected in each droplet. We used NCBI taxonomy for classification. Phage species whose maximum frequency of detected CDS length in each droplet is less than 0.4 were not displayed.

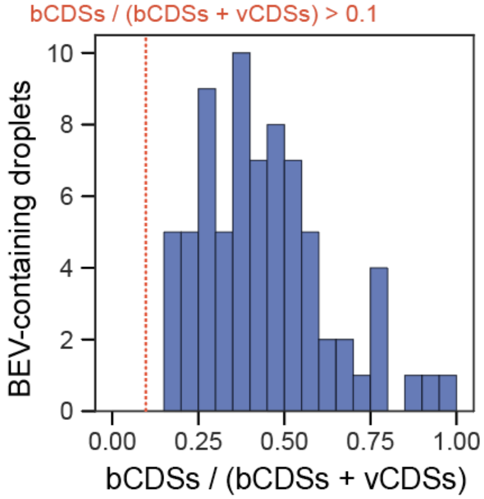

Supplementary Figure 15. The ratio of bCDSs to the sum of bCDSs and vCDSs counts in each droplet regarded as BEV-containing in the NP-DS of oral biofilm. The histogram of bCDSs / (bCDSs + vCDSs) in the BEV-containing droplets (bCDSs ≧ 5) (N = 73).

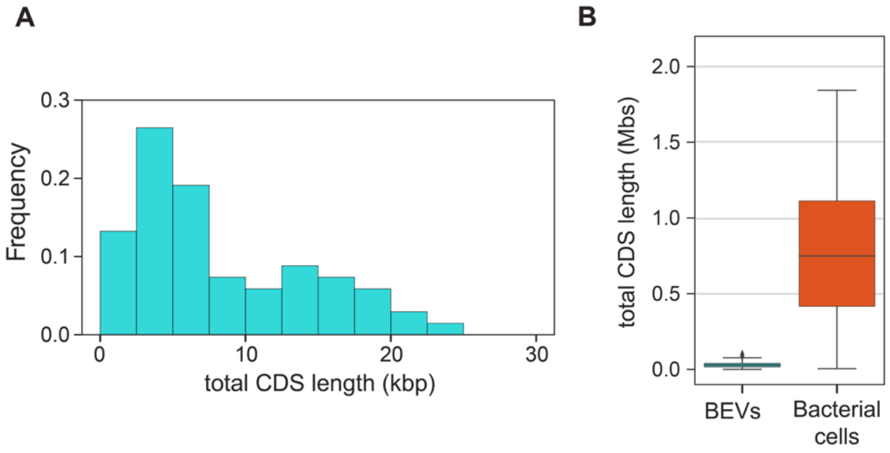

Supplementary Figure 16. Total detected CDS length in BEV-containing droplets analyzing oral biofilm. (A) The distribution of total length of detected bCDS region in BEV-containing droplets. (B) Box plots of total bCDS length in BEV-containing droplets (NP-DS) and bacterial cells containing droplets (cell-DS). Medians and outliers were shown as gray lines and diamonds each.

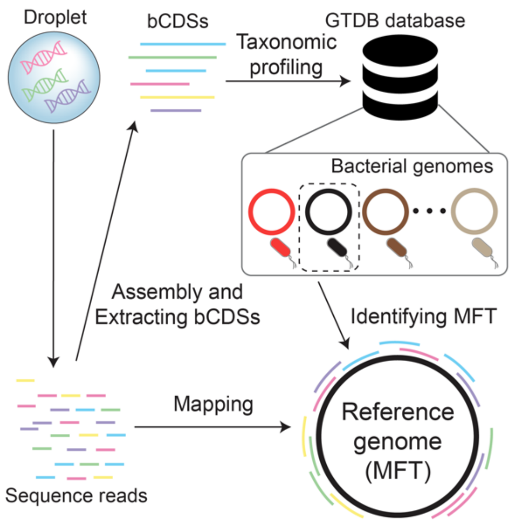

Supplementary Figure 17. A schematic of the analysis for identifying MFT and mapping on the reference genome. For each droplet, the most frequently detected bacterial taxon (MFT) was identified in GTDB taxonomy based on the annotated information of bCDSs (for details, see Materials and Methods). Then the read sequences were mapped to the corresponding assembly genome of the MFT.

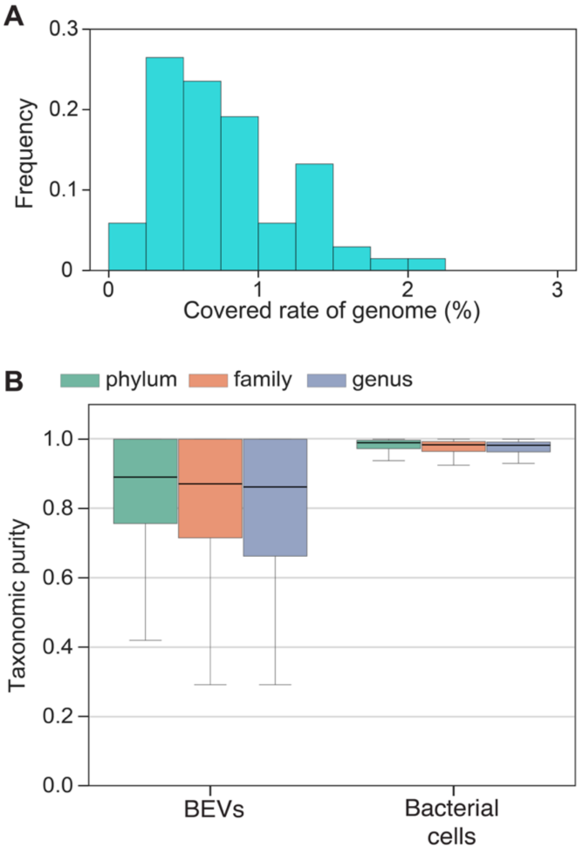

Supplementary Figure 18. Mapping status of sequence reads in BEV-containing droplets. (A) The distribution of the covered rate (The percentage of the genomic region covered by the sequence reads in a BEV -containing droplet, same as Fig. 5B). For each droplet, sequence reads are aligned to the assembled genome of the most frequently detected taxon (MFT in Supplementary Fig.17). (B) Taxonomic purity of detected CDSs in BEVs isolated from the dental plaque. Here, the most frequently detected taxon (MFT) to which BEV-derived CDSs were assigned was first determined, and then the percentage of CDSs length attributed to the MFT in each droplet was calculated and plotted as a boxplot (we call this parameter "purity" of CDSs). We quantified this parameter for the GTDB taxonomic profile in phylum, class, and genus levels. Medians and outliers were shown as gray lines and diamonds each.

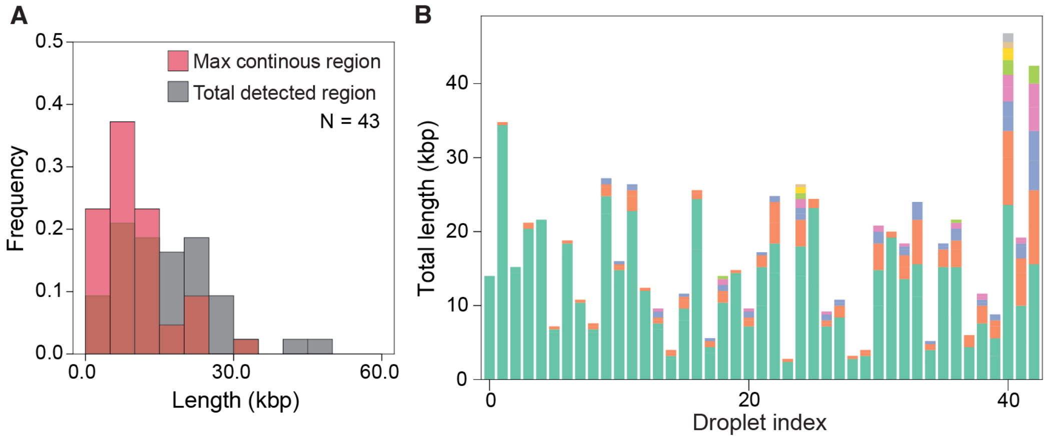

Supplementary Figure 19. The length of DNA fragments in the droplets containing *Alcaligenes faecalis* BEVs. (A) The distributions of the total lengths of the mapped genomic regions by sequence reads (gray) or the longest continuous genomic region mapped by sequence reads in each droplet (n = 43). The mapping profile is shown in Fig. 5C. (B) The lengths of detected contiguous genomic regions are plotted for each droplet containing *Alcaligenes faecalis* BEV. Stacked bar plots of different colors indicate DNA fragments originating from distant regions (> 2 kb) of the genome. Droplets are sorted in ascending order according to the ratio of the longest fragment length to the total detected region.

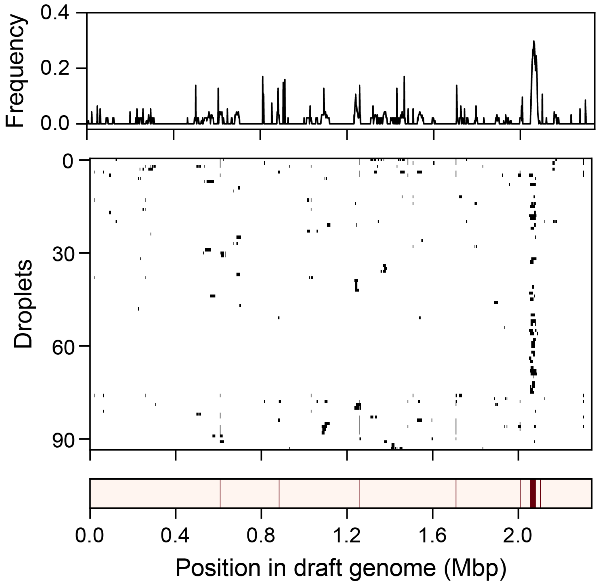

Supplementary Figure 20. Mapping profiles of sequence reads from *P. gingivalis* BEVs after cleaning of chimeric reads. The sequence reads were mapped after the cleaning process of chimeric reads using bwa. Same as Fig. 1B, the genomic positions where the sequence reads were mapped in each droplet were filled with black.

Supplemental Tables

Supplementary Table 1. Enriched CDSs in NP-DS of *P. gingivalis* BEVs. Enriched CDSs of *P. gingivalis* BEVs, which were screened using binomial test (*P* value < 0.01). Those screened genes were identical to those screened with the threshold of 75 percentile + 2*IQR (interquartile range).

Protein id	description	start position (bp)	end position (bp)
WP_013815260.1	hypothetical protein	2062054	2063016
WP_005874812.1	tetratricopeptide repeat protein	2057308	2058195
WP_005874932.1	hypothetical protein	2064777	2066783
WP_230456087.1	2TM domain-containing protein	2058359	2058541
WP_005874833.1	mechanosensitive ion channel	2056208	2057206
WP_230456086.1	T9SS type A sorting domain-containing protein	2058703	2059659
WP_005874913.1	hypothetical protein	2066813	2067403
WP_004583626.1	DNA-binding protein	2051440	2052066
WP_005874827.1	L-threonylcarbamoyladenylate synthase	2052478	2053053
WP_005874926.1	CRISPR-associated endonuclease Cas2	2069305	2069595
WP_005874927.1	hypothetical protein	2073529	2073972
WP_005874822.1	sugar transferase	2053050	2054456
WP_005874906.1	type III-B CRISPR module RAMP protein Cmr6	2072771	2073532
WP_005874912.1	type III-B CRISPR module-associated protein Cmr3	2074684	2075874
WP_010955945.1	IS5 family transposase	216542	217627
WP_005874916.1	CRISPR-associated endonuclease Cas1	2069615	2072710
WP_010956463.1	type III-B CRISPR module RAMP protein Cmr4	2073969	2074670
WP_010955941.1	IS4-like element IS1598 family transposase	199227	200384
WP_005873516.1	threonine-phosphate decarboxylase CobD	1239071	1240078
WP_005873512.1	cob(I)yrinic acid a,c-diamide adenosyltransferase	1241563	1242129
WP_005873514.1	cobyrinate a,c-diamide synthase	1242152	1243471
WP_012458603.1	DUF6261 family protein	2060211	2061263
WP_010956459.1	DUF6261 family protein	2063358	2064410
WP_010956028.1	IS5-like element ISPg8 family transposase	502077	503162
WP_005874030.1	MULTISPECIES: histidinol phosphate phosphatase	611806	612324
WP_005874035.1	purine nucleoside phosphorylase I, inosine and guanosine-specific	615600	616421
WP_005873715.1	histidinol-phosphate aminotransferase	623025	623531
WP_005874530.1	thiamine-phosphate kinase	689154	690194
WP_005873566.1	adenosylcobinamide amidohydrolase	696973	698136
WP_004342769.1	site-specific integrase	878721	879923
WP_004342772.1	hypothetical protein	879930	880292
WP_014709482.1	helix-turn-helix transcriptional regulator	881003	881272
WP_004584269.1	ABC transporter permease	1095538	1096284
WP_005873460.1	ATP-binding cassette domain-containing protein	1096303	1097037
WP_005873451.1	T9SS C-terminal target domain-containing protein	1097132	1098505
WP_004584372.1	S4 domain-containing protein	1236875	1237327
WP_010956235.1	adenosylcobinamide-phosphate synthase CbiB	1238047	1239057
WP_005874841.1	PH domain-containing protein	1374934	1375389
WP_004342775.1	nuclear transport factor 2 family protein	1534837	1535244

Supplementary Table 2. Enriched gene categories in NP-DS of *P. gingivalis* BEVs. Enriched GO terms in the enriched CDSs in the BEVs were statistically screened (hypergeometric distribution test, *P* value < 0.05). GO terms were grouped into three categories: M: Molecular functions; B: Biological processes; and C: Cellular components.

GO id	category	name	Members in original genome	Members in BEVs	*P* value
GO:0009236	P	cobalamin biosynthetic process	13	4	5.02E-05
GO:0048472	F	threonine-phosphate decarboxylase activity	2	2	0.000314
GO:0043571	P	maintenance of CRISPR repeat elements	4	2	0.00185
GO:0051607	P	defense response to virus	5	2	0.00304
GO:0006313	P	DNA transposition	11	2	0.0157

Supplementary Table 3. Enriched gene categories in bulk-BEV sequencing of *P. gingivalis*. Enriched GO terms screened in the bulk-BEV sequencing data (hypergeometric distribution test, *P* value < 0.05).

GO id	category	name	Members in original genome	Members in BEVs	*P* value
GO:0009236	P	cobalamin biosynthetic process	15	10	1.28E-06
GO:0015420	F	ABC-type vitamin B12 transporter activity	4	4	0.000243
GO:0043171	P	peptide catabolic process	6	4	0.00297
GO:0032259	P	methylation	10	5	0.00442
GO:0042026	P	protein refolding	4	3	0.00715
GO:0042351	P	'de novo' GDP-L-fucose biosynthetic process	2	2	0.0158
GO:0048472	F	threonine-phosphate decarboxylase activity	2	2	0.0158
GO:0000156	F	phosphorelay response regulator activity	2	2	0.0158
GO:0070009	F	serine-type aminopeptidase activity	2	2	0.0158
GO:0008641	F	ubiquitin-like modifier activating enzyme activity	2	2	0.0158
GO:0022857	F	transmembrane transporter activity	13	5	0.0164
GO:0006541	P	glutamine metabolic process	10	4	0.0276
GO:0009399	P	nitrogen fixation	3	2	0.0435
GO:0009408	P	response to heat	3	2	0.0435

Supplementary Table 4. A detailed information of terminal inverted repeats (TIRs) in or adjacent to the enriched gene clusters in PG or AF BEVs. The number of TIR corresponds to that shown in Fig. 2 or Fig. 5. Insert length indicates the length of the region flanked by each TIR.
The genomic positions of each TIR start and end sites are also shown.

species	cluster	TIR No.	Insert Length (bp)	TIR length (bp)	Left start (bp)	Left end (bp)	Right start (bp)	Right end (bp)
Porphyromonas gingivalis	Cobalamin biosynthesis	1	17961	14	1230980	1230993	1248941	1248954
Porphyromonas gingivalis	Cobalamin biosynthesis	2	17646	58	1231088	1231145	1248791	1248848
Porphyromonas gingivalis	Cobalamin biosynthesis	3	17354	76	1231205	1231280	1248633	1248708
Porphyromonas gingivalis	Cobalamin biosynthesis	4	11788	14	1236244	1236257	1248032	1248045
Porphyromonas gingivalis	Cobalamin biosynthesis	5	26730	15	1236763	1236777	1263493	1263507
Porphyromonas gingivalis	Cobalamin biosynthesis	6	26675	620	1237434	1238049	1264723	1265338
Porphyromonas gingivalis	Type III-B CRISPR	1	12979	15	2040184	2040198	2053176	2053190
Porphyromonas gingivalis	Type III-B CRISPR	2	15859	14	2041832	2041845	2057691	2057704
Porphyromonas gingivalis	Type III-B CRISPR	3	15278	14	2053628	2053641	2068906	2068919
Porphyromonas gingivalis	Type III-B CRISPR	4	19076	14	2060409	2060422	2079494	2079505
Porphyromonas gingivalis	Type III-B CRISPR	5	15929	14	2063556	2063569	2079494	2079505
Alcaligenes faecalis	O-antigen biosynthesis	1	27828	14	3282151	3282164	3309979	3309992

Supplementary Table 5. Enriched CDSs of *Alcaligenes faecalis* BEVs. CDSs detected in the enriched genomic region of *Alcaligenes faecalis* BEVs (P value < 0.01, binomial test). CDS regions were extracted using Prokka and searched against the reference protein fasta file.

Protein id	description	start position (bp)	end position (bp)
WP_042487686.1	glycolate oxidase subunit GlcF	981833	983053
WP_042487683.1	hypothetical protein	983361	984668
WP_009454582.1	zeta toxin family protein	1484000	1484545
WP_009454580.1	hypothetical protein	1484541	1484726
WP_042480022.1	helix-turn-helix transcriptional regulator	1484954	1485787
WP_042480881.1	efflux RND transporter periplasmic adaptor subunit	1896312	1897547
WP_042480884.1	ABC transporter ATP-binding protein	1897858	1898649
WP_042487215.1	3-(3-hydroxy-phenyl)propionate transporter MhpT	2690924	2692165
WP_042487212.1	hypothetical protein	2692500	2693114
WP_042486227.1	chlorohydrolase family protein	2981930	2983414

WP_042486230.1	tripartite tricarboxylate transporter substrate-binding protein	2983444	2984421
WP_042486802.1	aspartate/glutamate racemase family protein	3284430	3285188
WP_042486928.1	LysR substrate-binding domain-containing protein	3285492	3286376
WP_226791390.1	ABC transporter permease	3288210	3289034
WP_042486807.1	ABC transporter substrate-binding protein	3289037	3290047
WP_157766853.1	acyltransferase family protein	3290206	3292086
WP_054513292.1	Wzz/FepE/Etk N-terminal domain-containing protein	3292644	3293687
WP_042486816.1	exopolysaccharide biosynthesis polyprenyl glycosylphosphotransferase	3293751	3295019
WP_042486818.1	glycosyltransferase	3295032	3295835
WP_123794683.1	hypothetical protein	3295838	3296524
WP_054513305.1	IS5 family transposase	3296656	3297621
WP_054513289.1	hypothetical protein	3297683	3298210
WP_042486050.1	glycosyltransferase	3298210	3299310
WP_080723785.1	N-acetyl sugar amidotransferase	3299357	3300478
WP_042486045.1	AglZ/HisF2 family acetamidino modification protein	3300478	3301236
WP_042486042.1	imidazole glycerol phosphate synthase subunit HisH	3301236	3301850
WP_052362964.1	lipopolysaccharide biosynthesis protein	3301850	3303094
WP_042486039.1	DegT/DnrJ/EryC1/StrS family aminotransferase	3303137	3304228
WP_042486036.1	UDP-2-acetamido-3-amino-2,3-dideoxy-D-glucuronate N-acetyltransferase	3304228	3304815
WP_042486033.1	Gfo/Idh/MocA family oxidoreductase	3304815	3305759
WP_080723784.1	O-antigen ligase family protein	3305990	3307249
WP_042485889.1	LysR substrate-binding domain-containing protein	3379553	3380554
WP_042485886.1	tRNA 2-selenouridine(34) synthase MnmH	3380547	3381638
WP_042485881.1	NosR/NirI family protein	3381813	3384071
WP_042485877.1	TAT-dependent nitrous-oxide reductase	3384137	3386038
WP_226791393.1	nitrous oxide reductase family maturation protein NosD	3386049	3387386
WP_042485874.1	ABC transporter ATP-binding protein	3387373	3388281
WP_042485871.1	ABC transporter permease	3388288	3389115
WP_042485868.1	nitrous oxide reductase accessory protein NosL	3389115	3389627
WP_042485865.1	FAD:protein FMN transferase	3389663	3390667
WP_042485861.1	glucose-6-phosphate isomerase	3390760	3392328
WP_042485854.1	LTA synthase family protein	3393190	3394734
WP_042485851.1	SDR family NAD(P)-dependent oxidoreductase	3394727	3395497
WP_042485848.1	glycosyltransferase	3395487	3396956
WP_042485845.1	glycosyltransferase	3396975	3398057
WP_042485842.1	glycosyltransferase family 4 protein	3398057	3399370

Supplementary Table 6. Enriched gene categories of *Alcaligenes faecalis* BEVs. Enriched GO terms in the enriched CDSs in the BEVs of *Alcaligenes faecalis* were statistically screened (hypergeometric distribution test, *P* value < 0.05).

GO id	category	name	Members in original genome	Members in BEVs	*P* value
GO:0009243	P	O antigen biosynthetic process	9	5	9.30E-10
GO:0000271	P	polysaccharide biosynthetic process	4	4	1.44E-09
GO:0006065	P	UDP-glucuronate biosynthetic process	3	3	2.59E-07
GO:0009103	P	lipopolysaccharide biosynthetic process	15	3	0.000112
GO:0071555	P	cell wall organization	46	4	0.000198
GO:0000107	F	imidazoleglycerol-phosphate synthase activity	4	2	0.000258
GO:0000105	P	histidine biosynthetic process	14	2	0.00375
GO:0016829	F	lyase activity	18	2	0.0062

Supplementary Table 7. Oligonucleotide probes used for the quantitative real-time PCR analysis. For each target region, we used three complement oligonucleotide probes. FAM and TAMRA are the fluorescent labels used in this analysis, respectively.

Target region	Type	name
Region1	Forward	ATGAAGAAGTGGGCGGAATAG
Region1	Reverse	GCCTTACTGCGTCCAAAGA
Region1	Probe	[FAM]-TTCGATAAGCGGCGAAAGGGCTTC-[TAMRA]
Region2	Forward	AGGAAGCTGACGAAGCATAAG
Region2	Reverse	CTATCTCGGTGGCTCTCTATCA
Region2	Probe	[FAM]-TCCAAGCAGAATGACGATGACGCT-[TAMRA]

Supplementary Data 1 (separate file .xlsx format). Enriched CDSs in bulk BEV-sequencing of *P. gingivalis* BEVs. We screened genes whose FPKM were larger than the threshold (75 percentiles + 2*IQR (interquartile range).

Supplementary Data 2 (separate file .xlsx format). The list of terminal inverted repeats flanking (pseudo) transposase or site-specific integrase in the *P. gingivalis* W83 genome. We screened more than 9 bp of the

repeat sequences whose insertion length is more than 500 bp. The BEV count score shows the number of BEVs that contain the specific transposon.

Supplementary Data 3 (separate file .xlsx format). Homologous TIRs to those identified around the enriched genomic regions in BEVs of Porphyromonas gingivalis. The homologous TIRs to No.2 and No.3 TIRs shown in Fig. 2 (cobalamin biosynthesis gene cluster) were only identified. We screened the TIRs whose divergence score in RepeatMasker is less than 10, the length of TIR is longer than or equal to 14 bp and more than 60% of its reference TIR, and the insert length is more than 5 kb and less than 50 kbp. Droplet counts score indicates the average number of detected droplets in the target genomic region.

Supplementary Data 4 (separate file .xlsx format). A list of all possible sources of contaminant DNA. All the species information in GTDB and NCBI formats were based on the listed taxa in either of Salter *et al.,* 2014 or Poore *et al.,* 2020 [26, 27]. The DNA fragments that were taxonomically assigned to one of the listed accession numbers were eliminated.

Supplementary Data 5 (separate file .xlsx format). A list of genomes used for pangenome analyses of *Porphyromonas*. The bacterial genomes collected from GTDB (version r214) were subject to the completeness and contamination checks, resulting in 244 genomes that showed > 90% completeness and < 5% contamination.

SI References

1. Arikawa K, Ide K, Kogawa M *et al.* Recovery of strain-resolved genomes from human microbiome through an integration framework of single-cell genomics and metagenomics. *Microbiome*. 2021;**9**:202 https://doi.org/10.1186/s40168-021-01152-4
2. Hosokawa M, Nishikawa Y, Kogawa M *et al.* Massively parallel whole genome amplification for single-cell sequencing using droplet microfluidics. *Sci Rep*. 2017;**7**:5199 https://doi.org/10.1038/s41598-017-05436-4
3. Hosokawa M, Endoh T, Kamata K *et al.* Strain-level profiling of viable microbial community by selective single-cell genome sequencing. *Sci Rep*. 2022;**12**:4443 https://doi.org/10.1038/s41598-022-08401-y
4. Price AL, Jones NC, Pevzner PA. De novo identification of repeat families in large genomes. *Bioinformatics*. 2005;**21**:i351-i58 https://doi.org/10.1093/bioinformatics/bti1018
5. Siguier P, Perochon J, Lestrade L *et al.* Isfinder: The reference centre for bacterial insertion sequences. *Nucleic Acids Res*. 2006;**34**:D32-6 https://doi.org/10.1093/nar/gkj014
6. Krupovic M, Makarova KS, Forterre P *et al.* Casposons: A new superfamily of self-synthesizing DNA transposons at the origin of prokaryotic crispr-cas immunity. *BMC Biol*. 2014;**12**:36 https://doi.org/10.1186/1741-7007-12-36
7. Kono N, Arakawa K, Tomita M. Comprehensive prediction of chromosome dimer resolution sites in bacterial genomes. *BMC Genomics*. 2011;**12**:19 https://doi.org/10.1186/1471-2164-12-19
8. Parks DH, Chuvochina M, Waite DW *et al.* A standardized bacterial taxonomy based on genome phylogeny substantially revises the tree of life. *Nat Biotechnol*. 2018;**36**:996-1004 https://doi.org/10.1038/nbt.4229
9. Parks DH, Imelfort M, Skennerton CT *et al.* Checkm: Assessing the quality of microbial genomes recovered from isolates, single cells, and metagenomes. *Genome Res*. 2015;**25**:1043-55 https://doi.org/10.1101/gr.186072.114
10. Seemann T. Prokka: Rapid prokaryotic genome annotation. *Bioinformatics*. 2014;**30**:2068-9 https://doi.org/10.1093/bioinformatics/btu153
11. Emms DM, Kelly S. Orthofinder: Phylogenetic orthology inference for comparative genomics. *Genome Biol*. 2019;**20**:238 https://doi.org/10.1186/s13059-019-1832-y
12. Nguyen LT, Schmidt HA, von Haeseler A *et al.* Iq-tree: A fast and effective stochastic algorithm for estimating maximum-likelihood phylogenies. *Mol Biol Evol*. 2015;**32**:268-74 https://doi.org/10.1093/molbev/msu300

13. Kalyaanamoorthy S, Minh BQ, Wong TKF *et al.* Modelfinder: Fast model selection for accurate phylogenetic estimates. *Nat Methods*. 2017;**14**:587-89 https://doi.org/10.1038/nmeth.4285
14. Huerta-Cepas J, Serra F, Bork P. Ete 3: Reconstruction, analysis, and visualization of phylogenomic data. *Mol Biol Evol*. 2016;**33**:1635-8 https://doi.org/10.1093/molbev/msw046
15. Katoh K, Standley DM. Mafft multiple sequence alignment software version 7: Improvements in performance and usability. *Mol Biol Evol*. 2013;**30**:772-80 https://doi.org/10.1093/molbev/mst010
16. Capella-Gutierrez S, Silla-Martinez JM, Gabaldon T. Trimal: A tool for automated alignment trimming in large-scale phylogenetic analyses. *Bioinformatics*. 2009;**25**:1972-3 https://doi.org/10.1093/bioinformatics/btp348
17. Clarke GD, Beiko RG, Ragan MA *et al.* Inferring genome trees by using a filter to eliminate phylogenetically discordant sequences and a distance matrix based on mean normalized blastp scores. *J Bacteriol*. 2002;**184**:2072-80 https://doi.org/10.1128/JB.184.8.2072-2080.2002
18. Bansal MS, Kellis M, Kordi M *et al.* Ranger-dtl 2.0: Rigorous reconstruction of gene-family evolution by duplication, transfer and loss. *Bioinformatics*. 2018;**34**:3214-16 https://doi.org/10.1093/bioinformatics/bty314
19. Chen S, Zhou Y, Chen Y *et al.* fastp: An ultra-fast all-in-one FASTQ preprocessor. *Bioinformatics*. 2018;**34**:i884-i90 https://doi.org/10.1093/bioinformatics/bty560
20. Bankevich A, Nurk S, Antipov D *et al.* Spades: A new genome assembly algorithm and its applications to single-cell sequencing. *J Comput Biol*. 2012;**19**:455-77 https://doi.org/10.1089/cmb.2012.0021
21. Fu L, Niu B, Zhu Z *et al.* Cd-hit: Accelerated for clustering the next-generation sequencing data. *Bioinformatics*. 2012;**28**:3150-2 https://doi.org/10.1093/bioinformatics/bts565
22. Buchfink B, Xie C, Huson DH. Fast and sensitive protein alignment using diamond. *Nat Methods*. 2015;**12**:59-60 https://doi.org/10.1038/nmeth.3176
23. Kahlke T, Ralph PJ. Basta - taxonomic classification of sequences and sequence bins using last common ancestor estimations. *Methods Ecol Evol*. 2019;**10**:100-03 https://doi.org/10.1111/2041-210x.13095
24. Cock PJ, Antao T, Chang JT *et al.* Biopython: Freely available python tools for computational molecular biology and bioinformatics. *Bioinformatics*. 2009;**25**:1422-3 https://doi.org/10.1093/bioinformatics/btp163
25. Shen W, Ren H. Taxonkit: A practical and efficient ncbi taxonomy toolkit. *J Genet Genomics*. 2021;**48**:844-50 https://doi.org/10.1016/j.jgg.2021.03.006
26. Poore GD, Kopylova E, Zhu Q *et al.* Microbiome analyses of blood and tissues suggest cancer diagnostic approach. *Nature*. 2020;**579**:567-74 https://doi.org/10.1038/s41586-020-2095-1
27. Salter SJ, Cox MJ, Turek EM *et al.* Reagent and laboratory contamination can critically impact sequence-based microbiome analyses. *BMC Biol*. 2014;**12**:87 https://doi.org/10.1186/s12915-014-0087-z
28. Langmead B, Salzberg SL. Fast gapped-read alignment with bowtie 2. *Nature Methods*. 2012;**9**:357-U54 https://doi.org/10.1038/Nmeth.1923
29. Lasken RS, Stockwell TB. Mechanism of chimera formation during the multiple displacement amplification reaction. *BMC Biotechnol*. 2007;**7**:19 https://doi.org/10.1186/1472-6750-7-19
30. Li H, Durbin R. Fast and accurate long-read alignment with burrows-wheeler transform. *Bioinformatics*. 2010;**26**:589-95 https://doi.org/10.1093/bioinformatics/btp698
31. Kogawa M, Hosokawa M, Nishikawa Y *et al.* Obtaining high-quality draft genomes from uncultured microbes by cleaning and co-assembly of single-cell amplified genomes. *Sci Rep-Uk*. 2018;**8** https://doi.org/ARTN 205910.1038/s41598-018-20384-3
32. Boutet E, Lieberherr D, Tognolli M *et al.* Uniprotkb/swiss-prot. *Methods Mol Biol*. 2007;**406**:89-112 https://doi.org/10.1007/978-1-59745-535-0_4
33. Ashburner M, Ball CA, Blake JA *et al.* Gene ontology: Tool for the unification of biology. The gene ontology consortium. *Nat Genet*. 2000;**25**:25-9 https://doi.org/10.1038/75556
